# Amphiphilic Baskets for Supramolecular Nanoarchitectures at Interfaces: Inverted Monolayer Formation on Water

**DOI:** 10.1021/acs.langmuir.6c01742

**Published:** 2026-06-03

**Authors:** Tai Bowling-Charles, Nitesh Kumar, Sefa Ucar, Carson E. Ward, Shamma Jabeen Proma, Jovica Badjić, Heather C. Allen

**Affiliations:** † Department of Chemistry & Biochemistry, 2647 The Ohio State University, Columbus, Ohio 43210, United States; ‡ Materials Sciences Division, Lawrence Berkeley National Laboratory, 1 Cyclotron Road, Berkeley,California 94720, United States; § Faculty of Science, Department of Chemistry, Atatürk University, Erzurum 25240, Türkiye

## Abstract

Interfacial chemistry of molecular baskets remains poorly understood despite their promise for supramolecular applications of detection and sequestration of toxic molecules including those of illicit drugs, organophosphorus compounds, and anticancer agents. We present a fundamental investigation of the interfacial behavior of three amphiphilic supramolecular baskets (ASB 4, 8, and 12), having increasingly longer yet linear alkyl chains at the top of their bowl-shaped cavity. The studies were completed at the air–water interface to elucidate surface activity, interfacial stability, self-assembly, and monolayer organization that drive inverted monolayer formation, in which the molecular arms orient toward the aqueous phase in a configuration opposite to that typically observed for lipids. Herein, surface pressure–area isotherms of ASB 4, 8, 12, deposited on a water surface, were performed in tandem with nonequilibrium relaxation experiments to quantify surface activity, thermodynamic stability, and monolayer compressibility of the baskets’ monolayer assembly. Brewster angle microscopy enabled direct visualization of morphological evolution, aggregation, and packing at the interface. We show that systematic extension of the hydrocarbon arms, from four to 12 methylene groups, progressively modifies intermolecular packing, drives distinct two-dimensional aggregation pathways, and increases number densities at the air–water interface. Atomistic molecular dynamics simulations corroborate many of these experimentally observed trends and provide mechanistic detail on the cooperative roles of basket topology and interfacial concentration in regulating the hydration structure and dynamics within the cavities generated by surface-adsorbing baskets, consistent with observed variations in surface activity and packing. Our results establish how the topology of these unique supramolecules and their concentration govern interfacial organization and offer a rational framework for designing amphiphiles with predictable behavior at soft interfaces.

## Graphical abstract

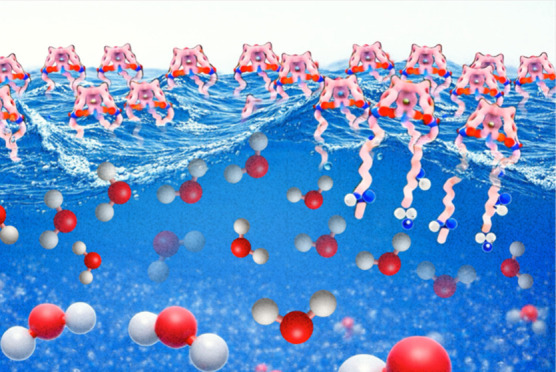

## Introduction

Molecular baskets are modular supramolecular hosts designed for inclusion complexation and recognition in both organic and aqueous environments.^1–4^ These bowl-shaped molecules feature a semirigid base, derived from tris-norbornene scaffold that extends into three aromatic sides, i.e. phthalimides, for encircling space.^1,2^ Functionalization of phthalimides achieved through a condensation with primary amines, allows incorporation of groups at the portal that may (a) improve solubility in water, (b) grant amphiphilic character, (c) prevent self-association and (d) regulate guest access (i.e., trafficking) to the basket’s cavity.^5^ With regard to the latest, the gating imparts responsiveness to environmental stimuli, enabling baskets to assume open or closed states and control guest trafficking.^1–3,6^ Importantly, molecular baskets have also been shown to trap biologically relevant molecules, including drugs of abuse, toxic organophosphorus compounds, and anticancer agents.^2,3,7^ These features position baskets as promising candidates for applications in the area of drug delivery and development of safe abiotic antidotes for detoxification.^1^

Despite significant prior research, little is known about organization of molecular baskets at the air–water interface, highlighting a critical knowledge gap. Recent studies have shown that nonclassical amphiphiles can display interfacial behavior controlled by molecular architecture, conformational adaptability, and supramolecular recognition rather than by hydrophobic packing alone. Core–shell bottlebrush “stealth surfactants” undergo architecture-driven inversion at liquid–liquid interfaces, where pH-responsive cores and hydrophobic shells reorganize to generate adaptive interfacial activity.^8^ Similarly, octadecyl acyclopa (ODA), a flexible amphiphilic ligand, forms pseudocyclic molecular complexes at organic/aqueous interfaces, demonstrating how conformational dynamics can influence interfacial recognition and selectivity.^9^ Amphiphilic cyanostar macrocycles further illustrate this concept, as anion binding converts the receptor into a charged supra-amphiphile that self-organizes into ordered monolayers at the air–water interface.^10^ In another study, stable, light-responsive Langmuir monolayers were shown to form from sterol–spiropyran conjugates, while separate work demonstrated that a macroscopic host molecule, ethoxy-functionalized pillar[5]­arene, also exhibits Langmuir isotherm behavior when cospread with a linear fatty acid.^11,12^ Finally, the broader field of nanoarchitectonics emphasizes that liquid interfaces provide versatile environments for organizing structures across a wide range of systems, from small molecules to polymers, nanosheets, proteins, and living cells.^13^

Within this context, the amphiphilic molecular baskets investigated in this work differ substantially from conventional amphiphiles because their interfacial behavior is governed not only by hydrophobic–hydrophilic balance, but also by their rigid three-dimensional supramolecular architecture. Classical amphiphiles, such as fatty acids, phospholipids, and simple surfactants, generally possess linear or cylindrical geometries that promote predictable interfacial packing through alkyl-chain interactions. In contrast, the ASB systems contain a concave basket-shaped scaffold with multiple alkyl arms, producing a sterically demanding and nonplanar topology that introduces additional geometric constraints during interfacial organization. Previous work from Badjić et al. showed that related amphiphilic baskets adopt truncated cone-like conformations, contain preorganized hydrophobic cavities, and can assemble into vesicular structures capable of organophosphonate recognition.^2^ These features distinguish ASBs from classical amphiphiles and support their potential as architecturally complex interfacial systems for supramolecular recognition at soft interfaces.

Molecular recognition at interfaces plays a central role in many biological processes yet remains less understood than bulk solution behavior. Interfacial environments can enhance effective molarity and orientational bias, thereby influencing recognition relative to bulk phases.^14^ Understanding their interfacial properties could enable the design of surface-active baskets capable of facilitating molecular transport across cell membranes and/or targeted sequestration of guest molecules. The chemistry and structure of surfaces, particularly the air–water interface, are fundamentally distinct from those of bulk aqueous phases. Reduced coordination, broken symmetry, and anisotropic solvation at the interface generate a molecular environment that differs markedly from the homogeneous liquid. For host–guest supramolecular systems, the air–water interface can act as both a concentrator and an orienting driver, increasing effective molarity, biasing guest approach geometries, and shifting thermodynamic binding parameters.^15,16^ This behavior is particularly important for macrocyclic hosts such as molecular baskets, cyclodextrins, cucurbiturils, calixarenes, or simple ether based crowns, whose surface activity and hydrophobic/hydrophilic balance can be tuned to control guest capture, release, and selectivity.^17–19^ Interfacial monomolecular films have been readily used as models for biomembranes and are locations of numerous biologically important interactions.^20–22^ By systematically tuning surface activity across a series of molecules, each more amphiphilic than the last, we uncover how subtle structural modifications influence interfacial adsorption, organization, packing density, and molecular orientation, leading to inverted monolayer formation at these bespoke interfaces.

To this end, we combined surface pressure methods, area isotherms, spectroscopy, microscopy, and all-atom molecular dynamics simulation to understand the interfacial chemistry of these unique supramolecular scaffolds, necessary to establish fundamental design principles for supramolecular applications ranging from biomimetic surface coatings to controlled drug delivery. In our investigation, we reveal that the tailored lengths and topology of hydrocarbon arms impact the interfacial adsorption and orientation preferences of the molecular baskets at the air–water interface, while their interfacial concentration further controls lateral organization and packing density, thereby modulating the structure of interfacial water. Specifically, longer aliphatic chains at the basket’s portal promote stronger interfacial adsorption and more effectively modulate the local hydration environment, resulting in the formation of nanoscopic cavities. We demonstrate how subtle modifications in molecular design can tune interfacial properties and optimize the functionality of cavity-containing supramolecular systems.

## Experimental Section

### Amphiphilic Supramolecular Basket Synthesis and Design

Amphiphilic supramolecular baskets (ASB 4, 8, and 12, Figure 1) hold three ammonium groups at their portal, each with four, eight or 12 methylene groups. These compounds were synthesized following the strategy outlined in Figure 1 (SI, Schemes S1 and S2). Briefly, the condensation of an amino alcohol with trivalent phthalic anhydride (i.e., *tris*-anhydride) afforded a trivalent alcoholic intermediate, which was subsequently mesylated to introduce an effective leaving group. Nucleophilic substitution with bromide in acetonitrile, followed by reaction with N-ethyl-N,N-dimethylamine, yielded ASB 4, 8, and 12. As for NMR spectroscopic characterization of newly prepared baskets (SI), *C*_3v_-symmetry is maintained for each amphiphilic compound.

**Figure 1:**
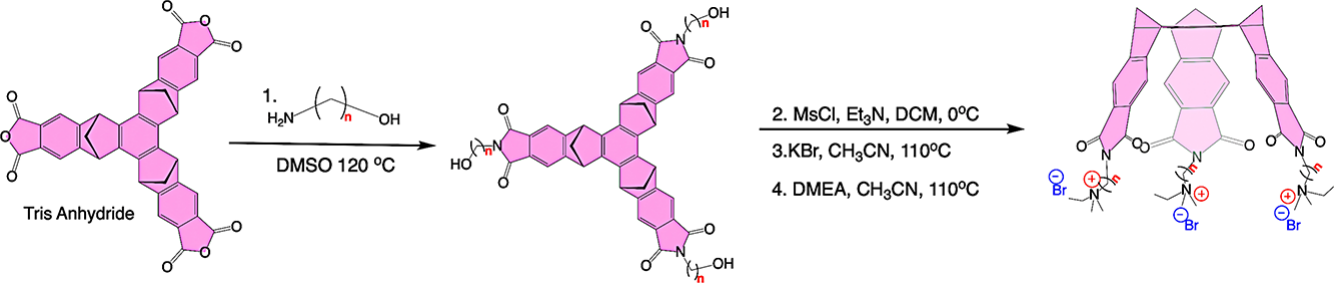
Synthetic route for amphiphilic supramolecular baskets ASBs-*n*, where *n* = 4, 8, or 12 denotes the alkyl arm length.

### Sample Preparation

Solutions of ASB dissolved in chloroform (HPLC grade, Fisher) were prepared in the concentrations of 0.1 mM (ASB-4, ASB-8, and ASB-12) and 0.01 mM (ASB-12). To limit contamination of the solvent, a sterile syringe and needle were used to transfer chloroform from the bottle directly into vials. These vials were thoroughly rinsed with water from a nano pure water system then rinsed with organic solvent. After the final water rinse, the vials were immersed in acid solution (98% concentration) for 24 h then dried in a 150 °C oven for at least 15 h before use. Each sample was sealed and placed in the sonicator for 30 s.

### Surface Pressure Area Isotherms and Non-Equilibrium Relaxation (**NER**)

The excess free energy at the air–water interface arises from the distinct molecular environments experienced by species at the surface relative to the bulk. Surface pressure Π is defined as the difference between the surface tension of the bare subphase γ_0_ and that of the monolayer covered surface γ. This quantity provides direct insight into molecular organization and structural evolution at the interface, with additional theoretical assessment in the SI.^23^

Surface pressure (Π) was measured as a function of mean molecular area (MMA) using a platinum Wilhelmy plate connected to a Teflon Langmuir trough (KSV NIMA) with an attached tensiometer and Delrin barriers (KSV NIMA). The barriers and trough were sequentially cleaned with reagent alcohol (Histological grade, Fisher Chemical) followed by ultrapure water. Prior to each experiment, the surface cleanliness was tested by filling the trough with the aqueous subphase and symmetrically compressing the barriers to check for any significant rise in surface pressure (≤0.20 mN/m). Using a micro syringe (50 μL, Hamilton), the 0.1 mM (ASB-4, ASB-8, and ASB-12) and 0.01 mM (ASB-12) basket solution was spread dropwise onto the aqueous subphase, and 15 min were allowed for the chloroform to evaporate after spreading. The Π-A isotherm compression occurred at a rate of 2 mm/min/barrier. Each measurement was taken at ∼19.5 °C and 30% relative humidity. The Π-A isotherm and NER measurements were taken in triplicate. We used a dilute (0.01 mM) solution of ASB-12 to monitor NER observations. (Prior titration studies showed that dilute concentrations facilitated reduction in molecular basket aggregation.^6,24^ ) For NER experiments, if there is a change in surface pressure upon monolayer relaxation, the barriers accommodate to offset the change with a set compression rate of 1 mm/barrier/s. This maintains a constant surface pressure, albeit the surface area reduces as the film relaxes.

### Infrared Reflection Absorption Spectroscopy (**IRRAS**)

*IRRAS* was employed as a surface sensitive technique to probe the organization of methylene and methyl groups along the hydrocarbon arms of the baskets. Spectra are reported as reflectance absorbance and were collected using unpolarized light in single beam mode, averaged over 400 scans. Reflectance absorbance is defined as the negative of the logarithmic ratio of the monolayer reflectance relative to the subphase reflectance, given by eq 1

$$\mathrm{RA}=-\log\left(\frac{R_{c}}{R_{0}}\right)$$ 1

where *R*_c_ corresponds to the reflectance of the monolayer covered surface and *R*_0_ denotes the reflectance of the bare subphase.^25,26^

IRRAS was performed using a Fourier transform infrared spectrometer (Spectrum 100, PerkinElmer) equipped with a liquid nitrogen-cooled HgCdTe (MCT) detector. Inside the spectrometer open cavity, the incident IR beam was controlled by a planar gold mirror at a 48° angle of incidence relative to surface normal of the solution. The reflected light was redirected toward the detector with a second gold mirror. The sample stage was placed under two gold mirrors, and a 57 mm glass Petri dish (with the subphase solution) was placed on top. Energy values were recorded every 0.5 cm^–1^ between 450 and 4000 cm^–1^, and the spectral resolution was 4 cm^–1^. Each experiment was repeated at 21.0 ± 2 °C and 30% relative humidity. The spectra were analyzed using OriginLab (2025b). Each experiment was repeated, and the standard deviation in peak position across trials was less than 0.1 cm^–1^.

### Brewster Angle Microscopy (**BAM**)

BAM allows real-time visualization of Langmuir monolayers.^27^ BAM images were acquired over a 2–3 h period at 30% relative humidity and a temperature of 19.5 ± 2 °C. BAM images demonstrated consistent domain shapes and morphological patterns, confirming the temporal stability of surface monolayers. The BAM images were processed using NIH public domain java imaging process software, ImageJ, (version 1.54f) and cropped from their original size to portray only the most resolved regions.

### Computational Protocol

To understand the nanoscopic chemistry governing adsorption, orientational ordering, and self-assembly of amphiphilic molecular baskets at the air–water interface, we constructed fully explicit biphasic simulation cells containing a liquid water slab with molecular baskets at the interface in equilibrium with an air phase. Initial configurations were generated using Packmol, which placed water molecules in the central region of the simulation box and randomly distributed baskets with unconstrained orientations in narrow regions adjacent to the upper and lower air–water interfaces, as illustrated in SI Figure S1a.^28^

The orthorhombic simulation domain spanned 60 × 60 × 180 Å in the *x*, *y*, and *z* directions, with the long dimension aligned perpendicular to the interface to allow the spontaneous formation of two well-separated air–water boundaries. Each system contained 7204 water molecules. To probe concentration-dependent adsorption and collective assembly, we embedded either 20 or 50 molecular baskets with hydrophobic arm lengths of four, eight, and 12 methylene units, partitioned at the two interfaces. The 60 × 60 Å lateral cell with 10 baskets per interface yields a simulated mean molecular area of 360 Å^2^/molecule, closely matched to the experimental coverage at which IRRAS and BAM measurements were performed (MMA_exp_ ≈ 470 Å^2^/molecule). The higher-loading system with 25 baskets per interface (MMA_sim_ = 144 Å^2^/molecule) is ∼3-fold denser than the experimental film and probes the compression-induced lateral organization analogous to the 3D-aggregate configuration. The two simulated loadings therefore bracket the experimentally accessible coverage and resolve the progression from spread-monolayer adsorption to crowding-driven assembly. In total, six different biphasic systems were simulated, comprising three basket variants (*n* = 4, 8, 12) at two surface loadings (20 and 50 baskets per box). Charge neutrality was preserved by solvating an appropriate number of chloride ions within the aqueous phase. All-atom molecular dynamics simulations were performed with the GROMOS-54A7 force-field for the baskets, and the SPC/E model for water.^29,30^ Nonbonded van der Waals interactions were treated using a Lennard-Jones potential with a real-space cutoff of 12 Å. The choice of an extended 12 Å cutoff is essential for correctly capturing packing fluctuations and hydrophobic association within the low-dielectric air region.

Electrostatic interactions were evaluated using the Particle Mesh Ewald (PME) method with a real-space cutoff of 12 Å, Fourier grid spacing of approximately 1.2 Å, and a fourth-order B-spline interpolation scheme.^31^ This choice ensures that interfacial polarization, image charge effects, and long-range dipolar correlations are represented with quantitative fidelity. The systems were propagated with a 2 fs time step using the leapfrog integrator in GROMACS-2023.4 package.^32^ Hydrogen bond lengths were constrained with LINCS, which allows stable integration at the chosen time step even for extended arm conformational dynamics. Temperature was maintained at 298 K using the velocity-rescale thermostat with a coupling constant of 0.1 ps, providing a gentle stochastic modulation compatible with realistic interfacial capillary modes. All simulations were run in the canonical (NVT) ensemble with fixed box dimensions. Periodic boundary conditions were applied in all directions, but the large vacuum region along *z* prevents spurious interactions between the two interfaces across periodic images.

Each system was simulated for a total of 200 ns, where initial 150 ns were designated as the equilibration phase.^33^ This interval also allows the amphiphilic supramolecular baskets to reorganize cooperatively, undergo arm rearrangement, and complete the slow collective processes associated with interfacial adsorption and lateral self-assembly.^34,35^ To verify equilibration, simulations of the *n* = 4 and *n* = 8 basket systems were extended to 300 ns, and the average basket distance to the nearest Gibbs Dividing Surface (GDS) was monitored as a sensitive order parameter for interfacial adsorption (SI Figure S2). After 150 ns the basket–GDS distance fluctuates around a stable plateau in both systems (∼2.30 ± 0.08 nm for *n* = 4; ∼1.59 ± 0.05 nm for *n* = 8), with no systematic drift over the additional 150 ns. Consecutive 50 ns block averages (150–200 ns vs 200–250 ns) agree to within 0.04 nm — well inside the within-block standard deviation — confirming that the interfacial population is statistically stationary by 150 ns.

Representative snapshots extracted prior to equilibration and following the emergence of the interfacial supramolecular layer are provided in SI Figure S1a,b to illustrate the evolution from a dispersed to an ordered interfacial state. Subsequent analysis focused exclusively on the final 50 ns of each trajectory. In this window the basket density profiles along the surface normal, the water density redistribution, the adsorption free energy landscapes, the orientational distributions defined by the angle between the interface normal and the vector from the center of mass of the central six-membered aromatic ring to the terminal arm nitrogen atoms, and the concentration dependent self-assembly patterns were studied.^36,37^

The Solvent-Accessible Surface Area (SASA) of the basket atoms was computed with the GROMACS gmx sasa utility.^38^ The total basket-group SASA at every saved frame, *A*_basket‑total_(*t*), was averaged over the analysis window and normalized to a per-atom intensive quantity by dividing by the number of basket molecules and the number of atoms per basket: ⟨*A*_per‑atom_⟩ = ⟨*A*_basket‑total_/(*N*_baskets_ × *N*_atoms/basket_). Reported uncertainties are the trajectory-frame standard deviation.

Translational exchange of water between the basket-O (O_ASB_) first solvation shell — defined by a cutoff of 0.45 nm corresponding to the first minimum of the O_ASB_–O_W_ pair correlation function (SI Figure S3) — and the bulk reservoir was quantified through the continuous shell survival probability, *S*_c_(*t*), defined as the fraction of waters tagged within the shell at a time origin that remain *uninterruptedly* in the shell up to lag *t*. The integral residence time was obtained by trapezoidal integration of *S*_c_(*t*) over the trajectory, τ_res_ = ∫_0_^*T*^ *S*_c_(*t*) d*t*, which corresponds to the mean first-passage time out of the shell.^39,40^

Reorientational dynamics of waters within the same O_ASB_ first solvation shell were characterized through the second-rank dipolar time correlation function,

$$C_{2}(t)=\langle P_{2}[{\hat{\mu }}_{i}(0)\cdot {\hat{\mu }}_{i}(t)]\rangle$$

where *P*_2_(*x*) = (3*x* ^2^ – 1)/2 is the second Legendre polynomial and μ̂_*i*_(*t*) is the unit vector along the HOH bisector of molecule *i*, constructed using minimum-image O–H vectors. The orientational relaxation time was obtained by trapezoidal integration of the correlation function up to its first zero crossing (t*), τ_rot_ = ∫_0_^t*^ *C*_2_(*t*) d*t*, which is the time scale most directly comparable to the rank-2 anisotropy probed by NMR and ultrafast IR pump–probe spectroscopies.^41^

## Results and Discussion

### Surface Pressure Area Isotherms

To elucidate how structural modifications shape interfacial behavior, surface pressure - area (Π-A) isotherms of ASB-4, ASB-8, and ASB-12 at the air- water interface were performed and are shown in Figure 2a. For ASB-4 and ASB-8, the surface pressure remains nearly zero upon barrier compression, a behavior that is particularly unexpected given their amphiphilic structures. The isotherm suggests that ASB-4 molecules absorb into the subphase or aggregates at the surface, although as we see below this is not entirely true.^10,42^ By extending the length of each arm by four carbons as in ASB-8, the minimal increase in the surface pressure is barely discernible, going from 0.00 to 0.15 mN/m, suggesting that increasing hydrocarbon arm length only slightly enhances surface activity.

**Figure 2:**
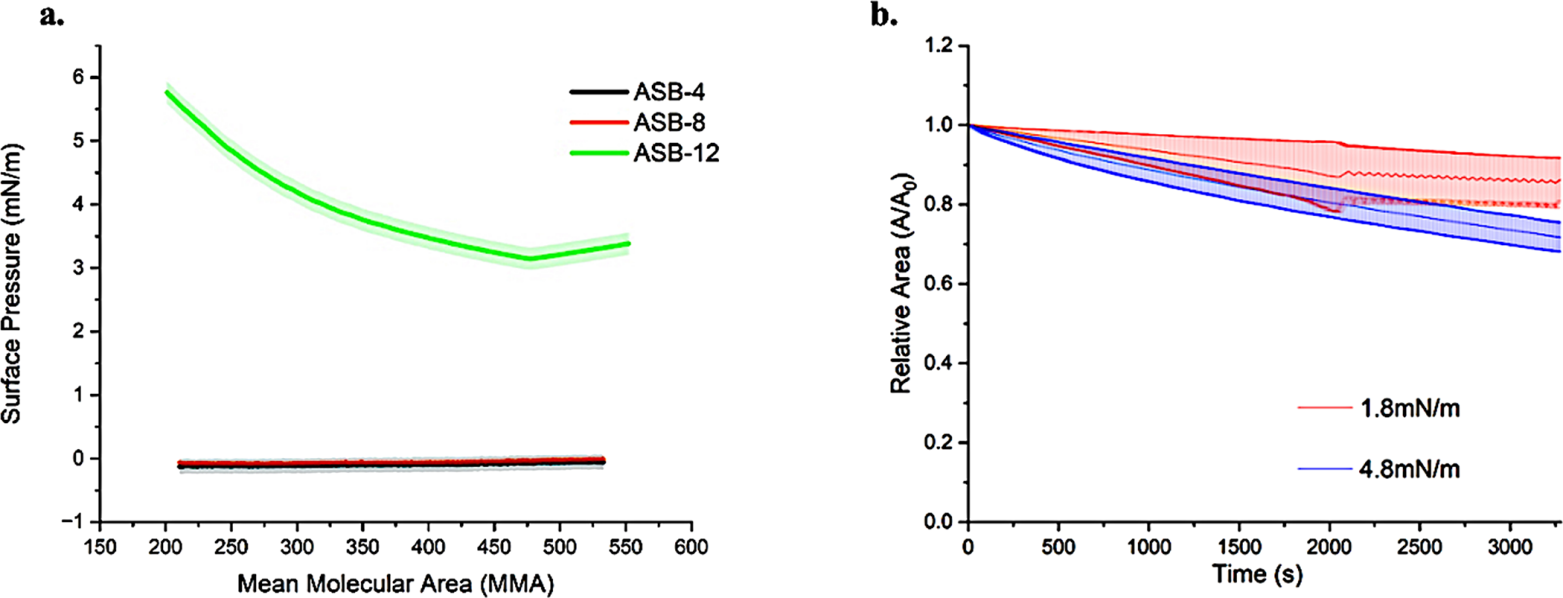
(a) Surface pressure–area isotherms of ASB-4, ASB-8, ASB-12 spread on neat water. Curves shown are the averages of three measurements. ASB-8 nearly overlaps with ASB4, although it is slightly higher in surface pressure. The shaded regions around the curves represent ± one standard deviation from the average three trials. (b) Monolayer NER curves of ASB-12 maintained at constant surface pressure of 1.8 mN/m (red) and 4.8 mN/m (blue). Curves shown are the averages of three measurements. The shaded regions around the curves represent ± one standard deviation from the average three trials. Each curve was calculated from the complete set of acquired data points without data reduction or smoothing. The relative area as a function of the log of time is plotted in SI Figure S6 to better highlight separation of the data sets.

To explore the effect of hydrocarbon arm length, we increased each arm by another four carbons to produce the ASB-12 basket. As shown in Figure 2a for ASB 12, the initial measurement exhibits a surface pressure of approximately 3.00 mN m^–1^ at 550 MMA. Upon initial compression from 550 MMA to approximately 480 MMA, an anomalous decrease in surface pressure is observed, followed by a distinct kink in the isotherm that indicates a phase transition, from a loosely packed — highly mobile layer — to a more ordered liquid-condensed phase, a behavior widely documented for saturated long-chain amphiphiles at the air–water interface.^20–22^ Upon further barrier compression, the isotherm followed a smooth nonlinear increase in surface pressure, suggesting a fluid—low order—rather than 2D crystalline, monolayer formation. The pronounced fluidity of the monolayer is evident from the isotherm, which lacks the sharp discontinuities typically associated with well-defined phase transitions.^20–22^ Comparatively, amphiphilic molecules such as lipids that have a polar headgroup oriented toward the water and a nonpolar tail facing the air, form monolayers with distinct phases at the air–water interface.^20^ Accordingly, prior studies of dipalmitoyl phosphocholine (DPPC) spread at the air–water interface and subjected to symmetric compression reveal four distinct phase transitions, corresponding to changes in lipid organization and orientation at the interface.^20–22^

As the ASB-12 film is compressed, its hydrocarbon arms begin to interact in a manner reminiscent of lipid tails, leading to a recognizable “lift-off” point—defined as the onset of measurable surface pressure beyond a few tenths of a mN/m.^23^ However, here we first observe measurable surface pressure, not quite meeting the lift off definition. In conventional surfactants such as DPPC, the headgroup is compact (∼1 nm), giving rise to well-defined lift-off behavior at smaller molecular areas.^43–45^ In contrast, the molecular baskets possess three hydrocarbon arms terminating in polar caps, which are expected to orient toward the aqueous subphase. This arrangement creates a larger effective interfacial footprint. For ASB-4, we estimate the cross-sectional area defined by the three arms to be approximately 193 Å^2^ supporting the expectation that these systems should display a higher lift-off area. Consistent with this prediction, ASB-4 and ASB-8 do not exhibit a distinct lift-off. However, ASB-12 shows a clear lift-off at ∼490 Å^2^, indicating that the longer arms promote sufficient lateral cohesion to initiate organized monolayer formation at the air–water interface.^23^ At the end of the compression, a collapse phase is not observed. Collapse would generally show a sharp decrease in surface pressure due to newly formed 3-D aggregates that do not disrupt the structure of water. However, some forms of cholesterol such as 7α-hydroxycholesterol or specific dumbbell shaped molecules such as amphiphilic bistable rotaxanes are known to undergo orientational adjustments when spread at the air–water interface in Langmuir films, thus, not exhibiting a true collapse.^46^

### Non-Equilibrium Relaxation (**NER**) Experiments

To test the stability of the ASB-12 layer at the air–water interface, nonequilibrium relaxation experiments were conducted. NER experiments involve compression to a specific surface pressure of the spread surfactant solution, and the target is maintained as the molecular area is monitored as a function of time.^47–50^ We used the relative area (*A*/*A*_0_) variations with time to obtain information on the relaxation mechanism of monomolecular films at the air–water interface.^51,52^ Generally, monolayer instability is common when films are spread to pressures above their equilibrium surface pressure. Instability can be caused by evaporation, film dissolution, intrinsic film rheology, and/or collapse of the film.^42,47^ We note that the equilibrium surface pressures are unknown for these molecular baskets. Here, we investigate the ASB-12 NER on neat water observing the change in relative area at 1.8 and 4.8 mN/m surface pressures.

At a constant surface pressure of 1.8 mN/m, ASB-12 exhibits a minimal film loss, which is indicated by the red (mostly) horizontal line in Figure 2b. Roughly less than 5% area loss is observed after 700 s. This ASB-12 monolayer reached a relative area of 0.86 after 3283 s. Initially, this system displays a slow initial decay then gradual increase over time. There is roughly 14% lost by 3272 s. This very slow decay with minimal area loss suggests a nucleation phase that involves structural rearrangement.^42,47^ Ultimately, the stability of the ASB-12 film at the surface decreases as the area percent loss increases to 14%. These results are comparable to a stearic acid monolayer stability study with various electrolytes in the subphase.^42,49^

The same experiment was conducted at a different surface pressure of 4.8 mN/m. At a constant surface pressure of 4.8 mN/m, there is an initial rapid area loss as observed within the blue curve in Figure 2b. There is greater than 5% area loss observed after only 500 s. Compared to the previous surface pressure of 1.8 mN/m, there is a clear initial decay then greater increase over time. By 3272 s, there is approximately 23% loss of area. This fast initial loss followed by greater area loss displays nucleation-type behavior.^47,51,52^ The NER curve was fit to the Vollhardt model of nucleation and growth (eq 2). This generalized equation helps identify the geometry of the nucleation centers and type of nucleation and growth.

$$\frac{\left(A_{0}-A\left(t\right)\right)}{\left(A_{0}-A_{\infty }\right)}=1-e^{-K_{x}t^{x}}$$ 2

In this expression, the initial monolayer area is represented by *A*_0_. *A*(*t*) is the total monolayer area at a specified time *t*, and at infinite time, the molecular area is represented by *A*_∞_. The exponent *x* is a quantitative marker used to determine the nucleation mechanism. Vollhardt describes four values (3/2, 5/2, 2, and 3) that are associated with various growths and nucleation geometry. *K*_*x*_ is the overall transformation constant.^47,48^ The first two terms, *A*(t) and *A*_0_, are obtained from the experimental data. *A*_∞_ is extrapolated from fitting the NER curve. First, a plot of *A*(t)/*A*_0_ versus the inverse of time is determined (SI Figure S4). When *t* = ∞, 1/*t* = 0; thus, the y-intercept of *A*(t)/*A*_0_ versus time will be *A*_∞_/*A*_0_. Rearranging eq 2 to an expanded form gives

$$\frac{A(t)}{A_{0}}=\left(1-\frac{\left(A_{0}-A_{\infty }\right)}{A_{0}}\right)+\left(\frac{\left(A_{0}-A_{\infty }\right)}{A_{0}}\right)e^{-K_{x}t^{x}}$$ 3

We calculated average *K*_*x*_ values for each *x* parameter from our experimental data then plotted the rearranged Vollhardt equation (eq 3) for all *x* parameters (SI Figure S5). The graph for *x* = 1.5 seems to fit the experimental data best. We therefore conclude from this value that the growth edge is hemispherical with instantaneous nucleation.^47–49^

### IRRAS Spectroscopy

To elucidate the organization of methylene and methyl groups along the hydrocarbon arms of the baskets, infrared reflection absorption spectroscopy was performed. As shown in Figure 3a (and SI Figures S7–S9, S11, show monolayer stability during IRRAS), spectra were collected after spreading ASB-4, ASB-8, and ASB-12 as monolayers at the air–water interface at a mean molecular area of 470 Å^2^ per molecule on neat water. From the spectra of ASB-4 and ASB-8, we do not observe distinguishable peaks, inferring that these molecular baskets may be more soluble and thus less surface active than anticipated. This is consistent with our prior surface pressure isotherm experiment (Figure 2a). Yet, as discussed further below, and elucidated in the BAM studies below, some ASB-4 and ASB-8 do exist at the surface of neat water.

**Figure 3:**
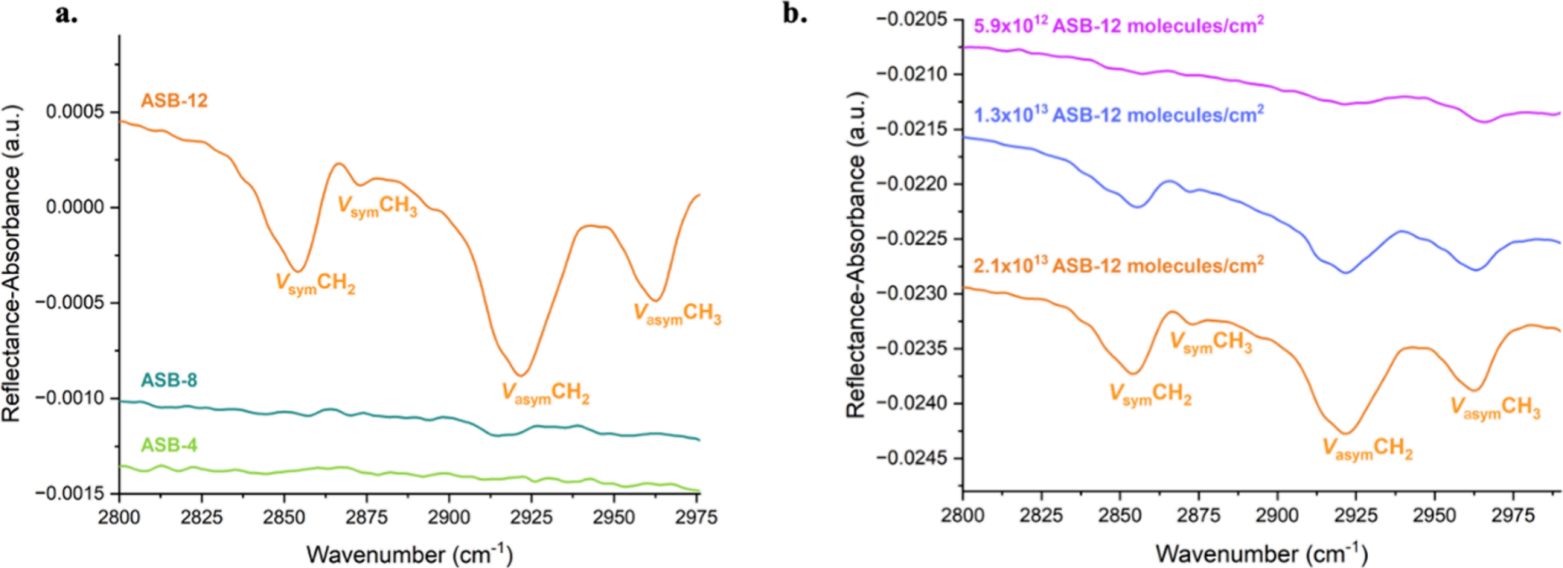
(a) IRRAS spectra in the CH stretching region of ASB-4, ASB-8, and ASB-12 on neat water (∼2.13 × 10^13^ molecules/cm^2^). (b) IRRAS spectra of various number densities of ASB-12 molecules on neat water in the CH stretching.

For ASB-12, we observe four distinct downward peaks from four different vibrational modes. The 2853 and 2921 cm^–1^ peaks are assigned to the CH_2_ symmetric and asymmetric stretch, respectively. The features at 2872 and 2962 cm^–1^ are attributed to the CH_3_ symmetric and asymmetric stretching modes, respectively. From Figure 3a, it can be deduced that there is a higher number density at the surface of neat water for ASB-12 (and SI Figures S10 and S11) compared to ASB-4 and ASB-8, despite that we spread the same number of molecules at their respective surfaces.^53,54^ In typical IRRAS spectra of lipid monolayers, the alkyl chain vibrational modes (such as the CH_2_ symmetric and asymmetric stretching bands) are not observed when the lipids are in the two-dimensional (2D) gaseous phase.^20,21,23,54^ This absence of signal occurs because the molecules are loosely packed and randomly oriented at the air–water interface, resulting in little to no net dipole change during vibration.^27,43,53,54^ To emphasize, the 2D gas-phase of DPPC is characterized by a molecular area greater than approximately 120 Å^2^ per molecule, a regime in which the alkyl chain modes become too weak to detect.^53–56^ Comparing this behavior to the IRRAS spectra of ASB-4 and ASB-8, we observe a similar lack of alkyl chain vibrational signatures. Based on this similarity of the ASB-4 and ASB-8 that reside at the aqueous surface, we can infer that the ASB-4 and ASB-8 hydrocarbon arms adopt similarly expanded monolayer states with low molecular packing density. In such a sparse and disordered arrangement, these molecules do not generate strong IRRAS signals in the alkyl region, consistent with a 2D gas-like phase analogous to that observed for DPPC.

The IRRAS peaks in the ASB-12 spectra are highly similar to those found in the liquid-expanded region of DPPC.^56^ We note that the alkyl chain of our molecular baskets is also similar to DPPC alkyl tails in structure, such that they are saturated and hydrophobic. We attribute our spectral observation to molecules undergoing restricted movement such that there is a transition from a 2-D gas-like phase to a 2-D liquid-like phase.^56,57^ A more well-ordered orientational distribution may also contribute to the observed signal intensity for the ASB-12 surface film.^54–56^

To examine the packing and orientation of the ASB-12 system, we collected IRRAS at various surface number densities. IRRAS is sensitive to conformational order of the alkyl chains and can inform on trans versus gauche chain conformations. Figure 3b lists the surface number density (molecules/cm^2^) for each spectral acquisition. For alkyl chains of lipids, gauche defects typically have vibrational CH_2_ asymmetric modes between 2924 and 2928 cm^–1^.^52^ The CH_2_ symmetric stretch has a peak between 2854 and 2856 cm^–1^.^52^ The CH_2_ symmetric peak is not distinguishable in the lowest surface number density spectrum of ASB-12 (Figure 3b, fuchsia spectrum). For higher surface number densities, the CH_2_ symmetric stretching peak shifts slightly from ∼2855 to 2853 cm^–1^. Although this 2 cm^–1^ red shift is close to the instrumental spectral resolution (1 cm^–1^), it is discernible. Red shifts (a shift to lower energy) observed for CH_2_ stretching bands are commonly associated with an increase in trans conformations and tighter alkyl chain packing.^26,54,58^

Additionally, we observe the CH_2_ asymmetric stretching peak in all samples of Figure 3b. At the lowest surface number density (fuchsia spectrum), this band is noticeably broadened, consistent with a larger population of disordered chains. As the surface number density increases, the peak becomes more defined and exhibits a small red shift from approximately 2922 to 2921 cm^–1^. Although the magnitude of the shift is small, the consistent directional change, combined with band narrowing, supports the interpretation of increased alkyl chain ordering.^26,54,58^ These subtle but reliable spectral trends highlight how the unique structure of the ASB-12 molecules influences monolayer packing behavior.

The CH_3_ symmetric peaks, also observed in Figure 3b, do not significantly shift with surface number densities. This peak is also not distinguishable in the lowest number density spectra (fuchsia data set). Finally, the CH_3_ asymmetric peak is also observed in each spectrum, and it red shifts from 2965 to 2963 cm^–1^ then to 2962 cm^–1^. Thus, we observed that with an increase of ASB-12 molecules on the surface of neat water, the methyl asymmetric and the methylene stretching modes red shift to lower wavenumbers. This observation indicates that there is an increase in trans configurations compared to gauche for the ASB-12 basket chains, and therefore, there is a higher degree of order at the higher number densities.^54,58,59^

### Brewster Angle Microscopy

We used BAM to visualize the organization of the monolayers, enabling direct observation of domain formation, aggregation, and packing behavior across the basket series. In Figure 4, each image was taken at 470 Å^2^ per molecule. Surprisingly, the BAM images of ASB-4 and ASB-8 revealed the formation of large two-dimensional aggregation structures at the air–water interface (Figure 4a,b). This was unexpected because both systems exhibited nearly zero surface pressure under compression, although there is a small detectable IRRAS signature for ASB-8 as well as a very small increase in surface pressure as shown above. In typical Langmuir monolayer behavior, surface-active amphiphilic molecules adsorb to the interface and become increasingly ordered during compression, resulting in a measurable rise in surface pressure.^18,20,25^ Thus, detectable surface pressure is often used as an indicator of molecular packing, film formation, and overall surface activity.^23,54^

**Figure 4:**
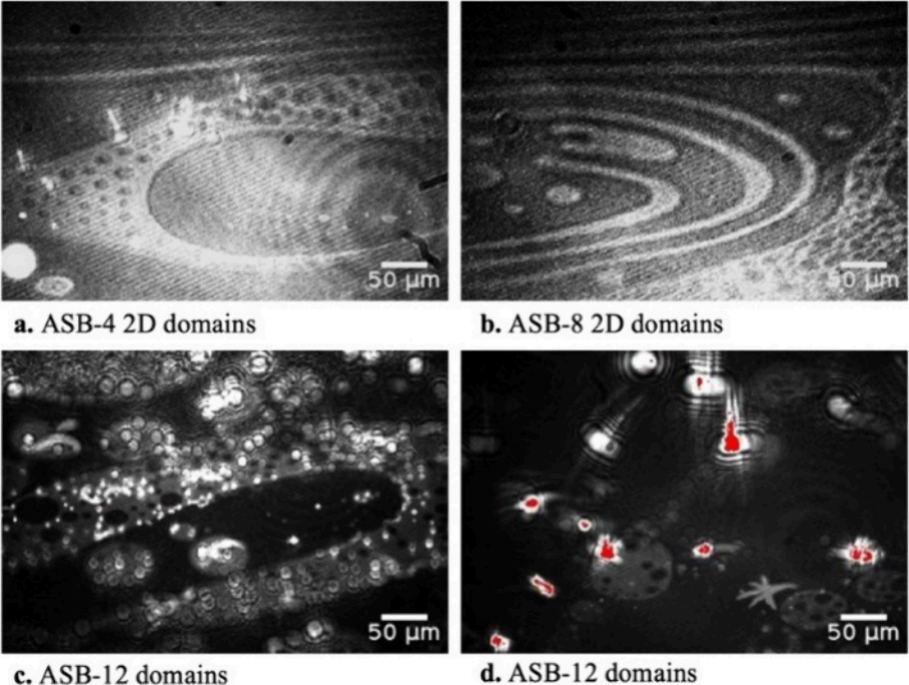
BAM Images of ASB-4 (a), ASB-8 (b), and ASB-12 (c and d) at 470 Å^2^ per molecule on water. (Red color areas in (d) are highest intensity regions such that the CCD camera pixels are near to saturation).

The absence of an increase in surface pressure for ASB-4 and ASB-8 would normally suggest minimal surface activity or weak lateral interactions;^10,20^ however, the BAM images clearly show that these molecules still associate and form 2D aggregates at the interface, indicating that surface pressure measurements alone do not fully capture their interfacial behavior. This demonstrates that even the smallest- hydrocarbon armed molecular basket (ASB-4) is surface active despite not producing a substantial surface pressure response. Interestingly, this behavior is reminiscent of palmitic acid monolayers, which display 2D aggregation in the gas–tilted condensed transition region (approximately 35 Å^2^ per molecule) before the lift-off point.^60^ The parallel between ASB-4/ASB-8 and a well-studied fatty acid system suggests that the ASB molecules may undergo structural or orientational reorganization at the interface that contributes to aggregation without an immediate rise in surface pressure. ASB-4 and ASB-8 form 2D domains due to the attractive intermolecular forces. Furthermore, there are morphological differences in the domain shapes as the hydrocarbon arm length is increased. Figure 4a,b have porous domains and membrane-like structures. Additionally, in Figure 4b, the structures are slightly more pronounced due to more light reflected off the film. ASB-8 has an increased number of CH_2_ groups in the hydrocarbon arms; therefore, more light is reflected because of greater packing or thickness level.^27^ The greater intensity observed for ASB-8 reflects a higher refractive index contrast caused by the additional CH_2_ units, which increases the optical density and thickness of the monolayer and thereby enhances light reflection.^27^ The dark pockets are predominately aqueous water regions where light is not reflected due to low or nonexistent number density of the basket molecules.^27,60,61^ In the images of ASB-12 (Figure 4c,d), there are 3D aggregates observed at the surface as highly reflective white and in some cases red (the red tint indicates camera sensor oversaturation, or error in camera refresh rate). Overall, each ASB system exhibits a mixture of large 2D domains structured similar to lipid membranes with only ASB-12 forming additional 3D aggregates on the surface. The elongated curved stripe-like morphologies in Figure 4 can be attributed to electrostatic dipole–dipole repulsions among the polar ends of the basket molecules.^59–62^ In addition, we attribute the formation of 3D aggregates to the self-aggregation of the longer arms in ASB-12. There are increased London dispersion forces among the CH arms, which can lead to entanglement of the arms. Together, the BAM images establish that hydrocarbon arm-dependent changes in optical contrast and intermolecular cohesion dictate the emergence of distinct 2D and 3D structures, revealing an interfacial landscape far richer than what is captured by surface pressure measurements alone.

BAM was also used to directly observe the varying systems of ASB-12 on neat water. Our IRRAS data (Figure 3b) informed us on the hydrocarbon arm conformation and ordering of the molecules at the air–water interfaces. In Figure 5, there is an increase in the number of structured domains as the number of molecules increases at the surface. There are more heterogeneous 2D domains present in 2.13 × 10^13^ molecules/cm^2^ (Figure 5c) of ASB-12 compared to the lesser volumes (Figure 5a,b). In the lowest volume (Figure 5a) there are nearly no distinguishable domain shapes present at the surface. As the number of molecules/cm^2^ increases, more membrane-like structures (consistent with the shapes previously observed in this study) appeared. Collectively, the data reveal a concentration-driven shift in ASB-12 from a diffuse interfacial film to structured domains.

**Figure 5:**
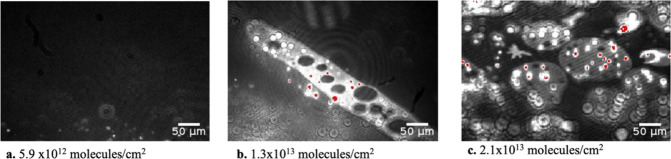
(a–c) BAM images of ASB-12 domains at various number densities (and thus various MMAs) on neat water. (Red color areas in (b) are highest intensity regions such that the CCD camera pixels are near to saturation).

### Molecular Dynamics Simulations

To quantify how the molecular baskets partition between the interfacial and bulk aqueous regions, we first computed one-dimensional number density profiles ρ_basket_(*z*) along the *z* axis for baskets with arm lengths n equal to 4, 8, and 12 in ASB-4, ASB-8, and ASB-12 (Figure 6a) respectively. These profiles reveal a clear increase in interfacial adsorption with increasing arm length. After equilibration, baskets with the shortest arms of four carbons exhibit a broad density distribution that extends well into the bulk aqueous phase, due to relatively high aqueous solubility and only a weak preference for the air–water interface (Figure 7), consistent with larger average basket distance from the nearest GDS for *n* = 4 compared to *n* = 8 (SI Figure S2b). As the arm length increases, the basket density develops a pronounced maximum within the interfacial region, indicating a stronger thermodynamic driving force for adsorption. The longer hydrophobic arms change the overall amphiphilicity of the baskets and effectively reduce their solubility in water, stabilizing configurations in which the aromatic cavity resides near the GDS while the charged arm remains solvated by the aqueous phase, as observed from a decrease in average Solvent Accessible Surface Area (SASA) per atom with basket chain length (SI Figure S12). For the ASB-12 system, the basket density is almost entirely localized within the interfacial region, in agreement with the experimental observations. Representative simulation snapshots show that these baskets adsorb with the charged arm buried in the aqueous phase and the aromatic headgroup oriented toward the air phase (Figure 7).

**Figure 6:**
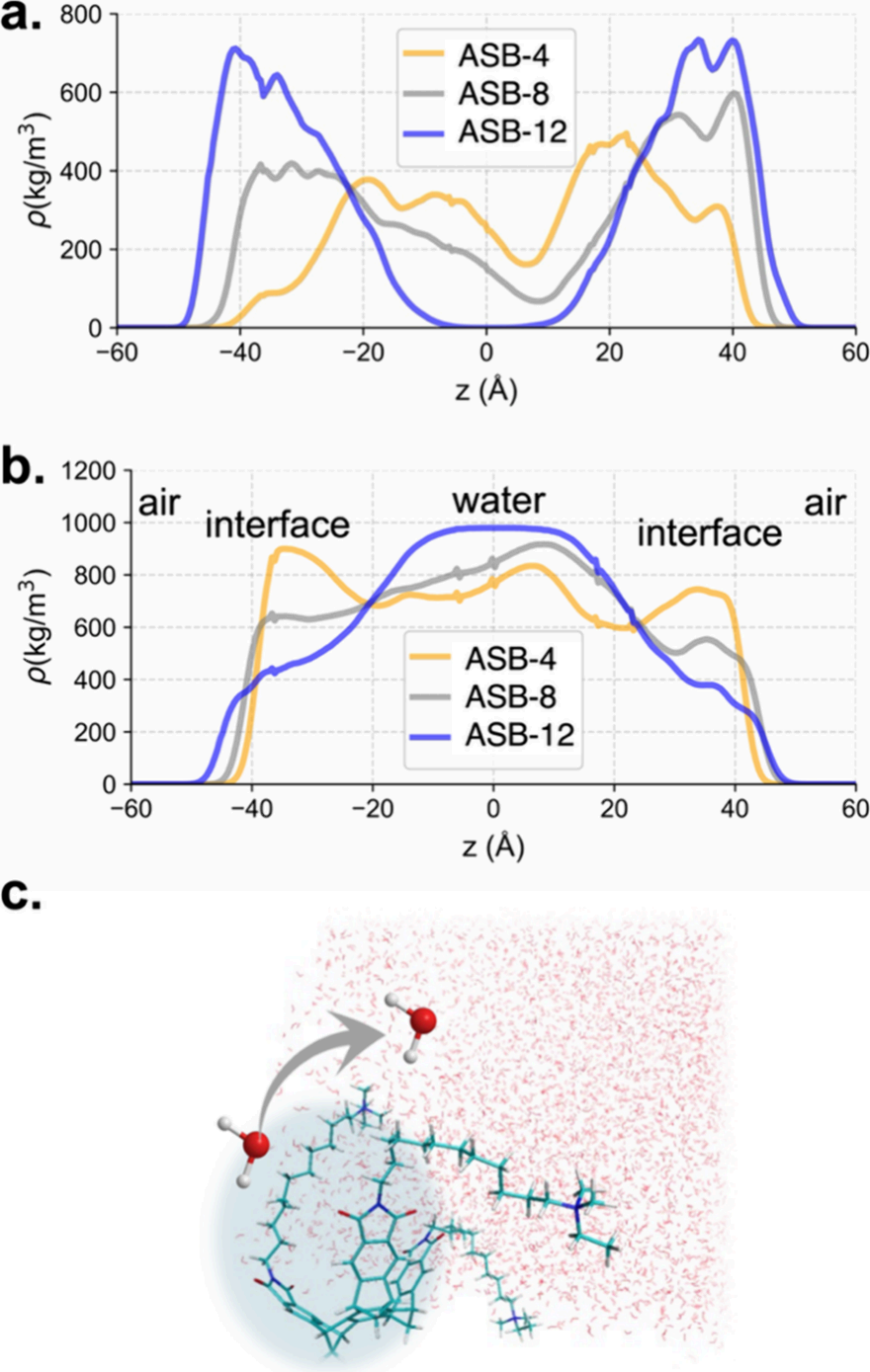
Molecular density profiles along the direction normal to the interface. Panel (a) shows the density of basket centers of mass for arm length four (ASB-4), eight (ASB-8), and 12 (ASB-12). Panel (b) shows the corresponding density of water oxygen atoms. Panel (c) presents a schematic illustration of interfacial water depletion and displacement induced by adsorption of the amphiphilic supramolecular baskets at the air–water interface.

**Figure 7:**
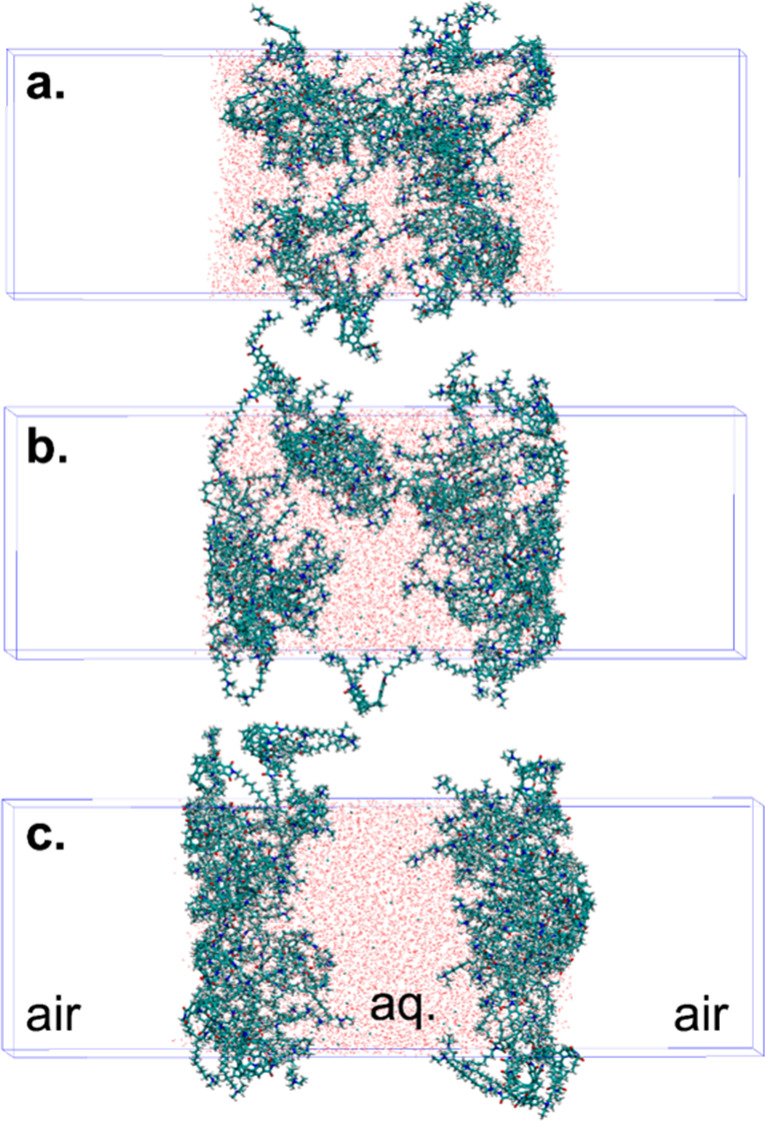
Representative molecular dynamics snapshots showing the equilibrium distribution of amphiphilic molecular baskets with hydrophobic arms of length (a) four, (b) eight, and (c) 12 methylene units at the air–water interface. Each panel is taken from the final portion of the trajectory and illustrates the partitioning of baskets between the interfacial and bulk aqueous regions. The wrapped version of the above simulation cells across the periodic boundary are provided in SI Figure S13.

Interestingly, the adsorption, orientation, and self-assembly of the ASB-12 baskets are strongly sensitive to the interfacial loading. At lower basket concentration, corresponding to 10 baskets per interface, the density profile along the *z* axis indicates that the adsorbed layer is confined to a relatively narrow region of about 20 Å (SI Figure S14e). Increasing the loading to 25 baskets per interface broadens this interfacial zone to roughly 40 Å, reflecting enhanced interfacial roughness and the onset of multilayer like packing. This change in vertical structure is accompanied by a qualitative change in lateral organization. At low loading, the baskets remain relatively dispersed on the air–water interface and form only small transient clusters, whereas at higher loading the probability of forming extended clusters increases substantially, consistent with the enlarged blue regions corresponding to low free energy (more favorable) in the two-dimensional free energy maps (SI Figure S14c,d). The same crowding driven transition also leaves a clear signature in the orientational ensemble. We quantify orientation by the angle between the interface normal and a vector connecting the center of mass of the central six membered aromatic ring to the terminal arm nitrogen atoms (SI Figure S15). At low concentration the distribution of this angle is relatively narrow and biased toward orientations that tilt the charged arm into the aqueous phase, while at higher concentration the distribution becomes much broader, indicating that packing constraints and basket–basket interactions generate a more heterogeneous spectrum of interfacial orientations.

The interfacial adsorption of the baskets strongly reorganizes the surrounding water. The water number density profile ρ_water_(*z*) exhibits a clear inverse correlation with the basket density ρ_basket_(*z*) (Figure 6b), as basket population accumulates within the interfacial region, the interfacial maximum in ρ_water_(*z*) is progressively attenuated and water is displaced from the immediate interfacial zone into the more bulk-like portion of the aqueous phase (Figure 6c), producing a pronounced trough near the air–water boundary that is compensated by an increase in ρ_water_ at larger *z*. Real-space visualizations show that this displacement generates nanoscopic cavities threaded between laterally associated baskets, yielding a highly heterogeneous microsolvation environment at the interface (SI Figure S14f). Quantitatively, this reorganization is reflected in the hydration of the basket polar sites themselves, where the average coordination number of water oxygens within 0.45 nm of any oxygen atom of a basket molecule decreases monotonically and substantially with arm length, from a total of 19.3 ± 5.0 for ASB-4 to 11.5 ± 5.8 for ASB-8 and only 6.5 ± 4.2 for ASB-12 (Figure 8a). The smaller, more aqueous-phase-resident ASB-4 baskets therefore retain a near-bulk hydration shell at their O sites, whereas the strongly interfacially adsorbed ASB-12 baskets present their polar oxygens to a depleted, partially dewetted environment in which roughly two-thirds of the bulk first-shell water population has been excluded. This systematic reduction in O-site hydration — a direct consequence of basket displacement away from the bulk subphase to adsorb into the interfacial layer — establishes the structural basis for the modified water dynamics discussed in the next section.

**Figure 8:**
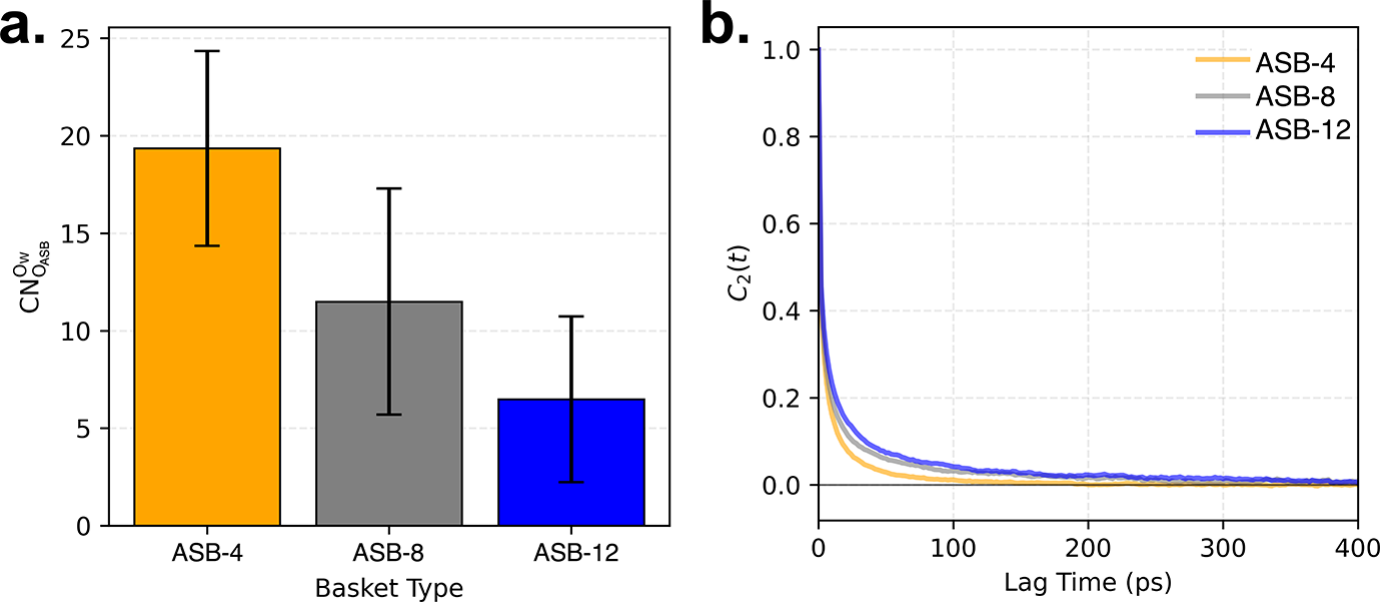
(a) Average coordination number of water oxygen atoms (O_W_) within 0.45 nm of basket oxygen atoms (O_ASB_), reported per ASB-4 (orange), ASB-8 (gray), and ASB-12 (blue) molecule. The 0.45 nm cutoff corresponds to the first minimum of the O_ASB_–O_W_ pair correlation functions. Error bars represent the standard deviation across the 50 baskets in each system, reflecting heterogeneity in local microsolvation environments at the interface. (b) Second-rank dipolar orientational correlation functions, *C*_2_(*t*), for water molecules within the first solvation shell of O_ASB_ of ASB-4 (orange), ASB-8 (gray), and ASB-12 (blue).

These nanoscopic cavities correspond to water depleted pockets that coexist with adjacent water rich regions and are stabilized by the cooperative packing of the basket arms. The extent of this microsolvation pattern depends sensitively on basket loading. For baskets with arm length ASB-12, the reduction in interfacial water density and the connectivity of the cavity network are markedly greater at the higher concentration per interface than at the lower loading (SI Figure S14e,f). Notably, the water that remains within these tailored environments is both translationally and orientationally constrained relative to bulk water. Quantitatively, the integral residence time of water within the basket-oxygen (O_ASB_) first solvation shell (τ_res_, obtained by integration of the continuous shell survival probability) increases monotonically with arm length, from 13.3 ps for ASB-4 to 15.4 ps for ASB-8 and 20.6 ps for ASB-12 (SI Figure S16); the orientational relaxation time of the bound waters (τ_rot_, obtained by integration of the dipolar correlation function *C*_2_(*t*)) follows the same ordering and shows an even larger spread, rising from 7.5 ps for ASB-4 to 14.7 ps for ASB-8 and 19.8 ps for ASB-12 (Figures 8b and S17). For reference, bulk SPC/E water at 298 K exhibits a reorientation time of approximately 2.5 ps, so even the most weakly perturbed (ASB-4) hydration shell is rotationally slowed by a factor of ∼3, while the ASB-12 shell is slowed by nearly an order of magnitude.^63,64^ The systematic dependence of both time scales on arm length demonstrates that basket topology controls not only the interfacial adsorption propensity and orientation of the baskets themselves but also the structure and dynamics of the interfacial water that they organize, producing a hydration environment that is increasingly distinct from bulk water as the hydrocarbon arms lengthen.

Overall, these results demonstrate that basket topology and interfacial concentration provide complementary handles to sculpt interfacial structure and hydration, offering a route to engineer host-guest binding, selective release, and separation processes that exploit finely tuned confinement and hydration thermodynamics at soft interfaces.

## Conclusions

In this work, we studied the interfacial chemistry of amphiphilic molecular baskets at the air–water interface by combining surface tensiometry, IRRAS, Brewster angle microscopy, and MD simulations. Our IRRAS measurements revealed that ASB-4 and ASB-8 display measurable surface activity, yet with significantly lower hydrocarbon arm ordering and interfacial number densities than ASB-12. The extended hydrocarbon arms in ASB-12 promote tighter alkyl packing, yielding a more stable and densely organized interfacial film. Surface pressure–area isotherms together with nonequilibrium relaxation experiments further show that ASB-12 forms a mechanically stable monolayer at low surface pressures and relaxes through nucleation and hemispherical growth at higher loadings, emphasizing its sensitivity to two-dimensional crowding and its capacity for structural reorganization. Brewster angle microscopy provides direct real time visualization of the influence of arm length on interfacial morphology. Despite generating negligible surface pressure, ASB-4 and ASB-8 form extensive two-dimensional structures, indicating lateral association driven by attractive intermolecular interactions. Domain morphology evolves from porous assemblies in ASB-4 to more membrane-like structures in ASB-8 and ultimately to thick two- and three-dimensional aggregates in ASB-12. These results demonstrate that even modest structural variations, such as a four-carbon increase in arm length, substantially alter interfacial packing, refractive contrast, and supramolecular organization.

Atomistic molecular dynamics simulations resolved the molecular origin of many of these trends. Extending the hydrocarbon arms drove progressive interfacial adsorption — with ASB-12 becoming essentially fully localized at the air–water boundary — and triggered a distinct transition from dispersed adsorption to lateral cluster formation at higher loadings, accompanied by broader orientational distributions and increased interfacial roughness. Basket adsorption simultaneously reorganized the surrounding hydration environment, displacing interfacial water into the bulk and generating a network of nanoscopic cavities between laterally associated baskets. The water that remained within these cavities was both translationally and rotationally confined, with residence and reorientation times slowed by nearly an order of magnitude relative to bulk water and scaling monotonically with arm length, showing a coupling between basket topology, interfacial assembly, and water-network reorganization that is inaccessible to the experimental observables alone. These combined experimental and computational results establish a unified framework for supramolecular basket organization into inverted monolayers at soft interfaces, providing fundamental insight and actionable design principles for engineering basket-based systems for biomimetic coatings, selective guest capture, and controlled release. Positioning amphiphilic molecular supramolecular baskets at fluid interfaces enables a new class of supramolecular hosts that function as binding sites at macroscopic boundaries.

## Supplementary Material



## Data Availability

Data including original raw spectra and processed data will be submitted to the Dryad database and are available upon request.

## Notes

NMR spectra and synthetic procedures for amphiphilic supramolecular baskets (ASBs); representative molecular dynamics snapshots of the molecular basket systems at the air–water interface; time evolution of surface pressure during adsorption experiments; determination of molecular area (*A*_∞_) from relative area vs reciprocal time (1/*t*) analysis; variability of the constant-pressure response of ASB-12 monolayers on water at 4.8 mN/m; surface pressure–area (π–*A*) isotherm measurements and experimental methodology; reproducibility of infrared reflection–absorption spectroscopy (IRRAS) measurements; semilogarithmic plots of the relative area relaxation data, generated from the complete data set, along with expanded *y*-axis views to better distinguish the ASB relaxation profiles; simulation snapshots; pair correlation functions; solvent-accessible surface area; ASB orientation distributions; density profiles; translational survival probabilities; and average water orientational relaxation times (PDF)
