# Supplementary material for: Amphiphilic Baskets for Supramolecular Nanoarchitectures at Interfaces: Inverted Monolayer Formation on Water
Source: Langmuir. 2026 Jun 3;42(23):16910–21. doi: 10.1021/acs.langmuir.6c01742 (PMC13276888; doi:10.1021/acs.langmuir.6c01742)
Supplement: Supplementary file 1 [file la6c01742_si_001.pdf]

Supplementary Information

Amphiphilic Baskets for Supramolecular Nanoarchitectures at Interfaces: Inverted Monolayer Formation on Water

Tai Bowling-Charles[a], Nitesh Kumar[b*], Sefa Ucar[c], Carson E. Ward[a], Shamma Jabeen Proma[a], Jovica Badjić[a], Heather C. Allen[a*]

[a] *Department of Chemistry & Biochemistry, The Ohio State University, Columbus, Ohio 43210, United States.*

[b] *Materials Sciences Division, Lawrence Berkeley National Laboratory, 1 Cyclotron Road Berkeley, CA 94720, United States.*

[c] *Atatürk University, Faculty of Science, Department of Chemistry, Erzurum 25240, Türkiye.*

Corresponding Authors

Nitesh Kumar, email: kmnitesh05@lbl.gov

Heather C. Allen, email: allen@chemistry.ohio-state.edu

Synthetic Procedures

Amphiphilic Supramolecular Basket 8 (ASB-8)

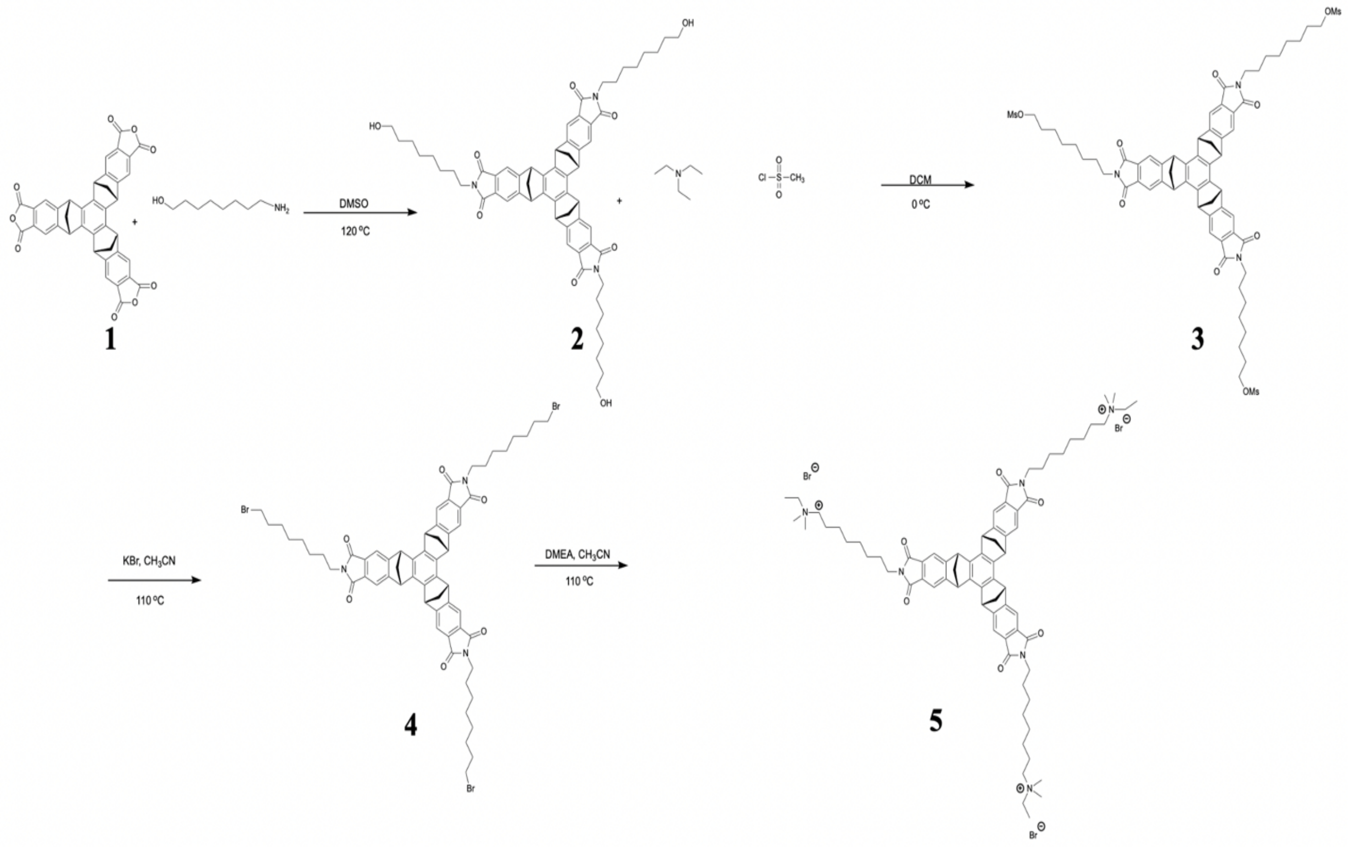

Scheme S1. Synthesis of ASB-8 (5). Supporting NMR data below.
For the preparation of 1, see Badjic et al. Molecules 2014, 19(9), 14292--14303.

Compound 2: To a suspension of tris-anhydride (1) (50 mg, 0.079 mmol) in DMSO (2 mL) was added 8-aminooctan-1-ol (34.55 mg, 0.238 mmol). The mixture was heated at 120°C for 4 hours and then solvent was removed by using a strong stream of nitrogen gas. The solid residue was purified by column chromatography (SiO2, CH2Cl2:CH3OH=30:1) to yield SBTN-(CH2)8-OH (55 mg, 68%). 1H NMR (400 MHz, Chloroform-d, 300 K) δ (ppm) = 7.46 (s, 6H), 4.46 (s, 6H), 3.53 (t, J = 6.6 Hz, 6H), 3.42 (t, J = 7.3 Hz, 6H), 2.54 (s, 6H), 2.17 (bs, 3H), 1.44 – 1.55 (m, 12H), 1.35 – 1.02 (m, 24H). 13C NMR (101 MHz, CDCl3) δ 168.34, 156.55, 137.89, 130.64, 116.04, 65.87, 62.86, 49.13, 40.92, 37.71, 32.64, 29.15, 28.89, 28.46, 26.60, 25.58, HRMS ESI: m/z calculated for $C_{63}H_{69}N_3O_9$: 1011.50 [M+H]+,found: 1012.51

Compound 3: To a solution of SBTN-(CH2)8-OH (50 mg, 0.049 mmol) in dry dichloromethane (5.0 mL) at 0°C was added triethylamine (60 mg, 0.593 mmol) and methanesulfonyl chloride (34 mg, 0.296 mmol). The mixture was stirred for 4 hours and water (10.0 mL) was added. After dilution with dichloromethane (30 mL), the organic phase was washed with water (20 mL) and brine (20 mL),and dried over sodium sulfate. Upon removal of the solvent, the solid residue was purified by column

chromatography (SiO_2,CH_2Cl_2:CH_3OH = 100:1) to yield SBTN-(CH_2)8-OMs (50 mg, 81%). 1H NMR (400 MHz, Chloroform-d, 300 K) δ (ppm) = 7.46 (s, 6H), 4.46 – 4.33 (m, 6H), 4.13 (t, J = 6.6 Hz, 6H), 3.42 (t, J = 7.3 Hz, 6H), 2.94 (s, 9H), 2.54 (t, J = 1.6 Hz, 6H), 1.64 (dt, J = 14.8, 6.6 Hz, 6H), 1.44 (dt, J = 13.8, 7.1 Hz, 6H), 1.35 – 1.07 (m, 24H) 13C NMR (101 MHz, $CDCl_3$) δ 168.30, 156.60, 137.89, 130.63, 116.03, 70.20, 65.87, 49.13, 37.63, 37.34, 29.01, 28.79, 28.73, 28.42, 26.54, 25.26; HRMS ESI: m/z calculated for $C_{66}H_{75}N_3O_{15}S_3$:1245.46 [M+H]+,found: 1246.44

Compound 4: To a solution of SBTN-(CH_2)8-OMs (50 mg, 0.040 mmol) in dry acetonitrile (5.0 mL) was added KBr (477 mg, 4.0 mmol). The mixture was heated in a sealed tube at 110°C for 10 hours. Solvent was removed under reduced pressure and solid residue was dissolved in dichloromethane (20 mL) and water (20 mL). The organic layer was washed with brine (30 mL) and dried over sodium sulfate. Upon removal of the solvent, the solid residue was purified by column chromatography (SiO_2, CH_2Cl_2:CH_3OH=80:1) to yield SBTN-(CH_2)8-Br (40.0 mg, 83%). 1H NMR (400 MHz, Chloroform-d, 300 K) δ (ppm) = 7.46 (s, 6H), 4.95 – 4.02 (m, 6H), 3.53 – 3.47 (m, 6H), 3.30 (t, J = 6.8 Hz, 6H), 2.64 – 2.56 (m, 6H), 1.80 – 1.72 (m, 6H), 1.56 – 1.47 (m, 6H), 1.41 – 1.31 (m, 6H), 1.30 – 1.19 (m, 18H). 13C NMR (101 MHz, $CDCl_3$) δ 168.32, 156.53, 137.89, 130.65, 116.05, 65.84, 49.13, 37.73, 33.97, 32.73, 28.85, 28.56, 28.48, 28.05, 26.64; HRMS ESI: m/z calculated for $C_{63}H_{66}Br_3N_3O_6$:1201.25 [M+H]+,found:1202.25

Compound 5: To a solution of SBTN-(CH_2)8-Br (40.0 mg, 0.0333 mmol) in dry acetonitrile (10.0 mL) was added N,N-dimethylethylamine (243 mg, 3.33 mmol). The mixture was heated in a sealed tube at 110°C for 10 hours and then concentrated under reduced pressure. The solid residue was recrystallized from CH_3OH:ethyl acetate=1:10 to yield SBTN-(CH_2)8-NEtMe2Br (40.0 mg, 85%). 1H NMR (400 MHz, DMSO-d6, 300 K) δ (ppm) = 7.76 (s, 6H), 4.74 (s, 6H), 3.37 (t, J = 7.2 Hz, 6H), 3.35 – 3.27 (m, 6H), 3.27 – 3.11 (m, 6H), 2.97 (s, 18H), 1.71 – 1.56 (m, 6H), 1.53 – 1.38 (m, 6H), 1.31 – 1.10 (m, 32H). 13C NMR (101 MHz, DMSO) δ 168.36, 158.15, 138.38, 130.18, 116.48, 65.62, 62.84, 60.23, 58.98, 48.70, 28.79, 28.67, 28.37, 26.60, 26.13, 22.02, 8.29; HRMS ESI: m/z calculated for $C_{75}H_{98}N_6O_6Br_3$:1420.36 [M+H],found:1420.99

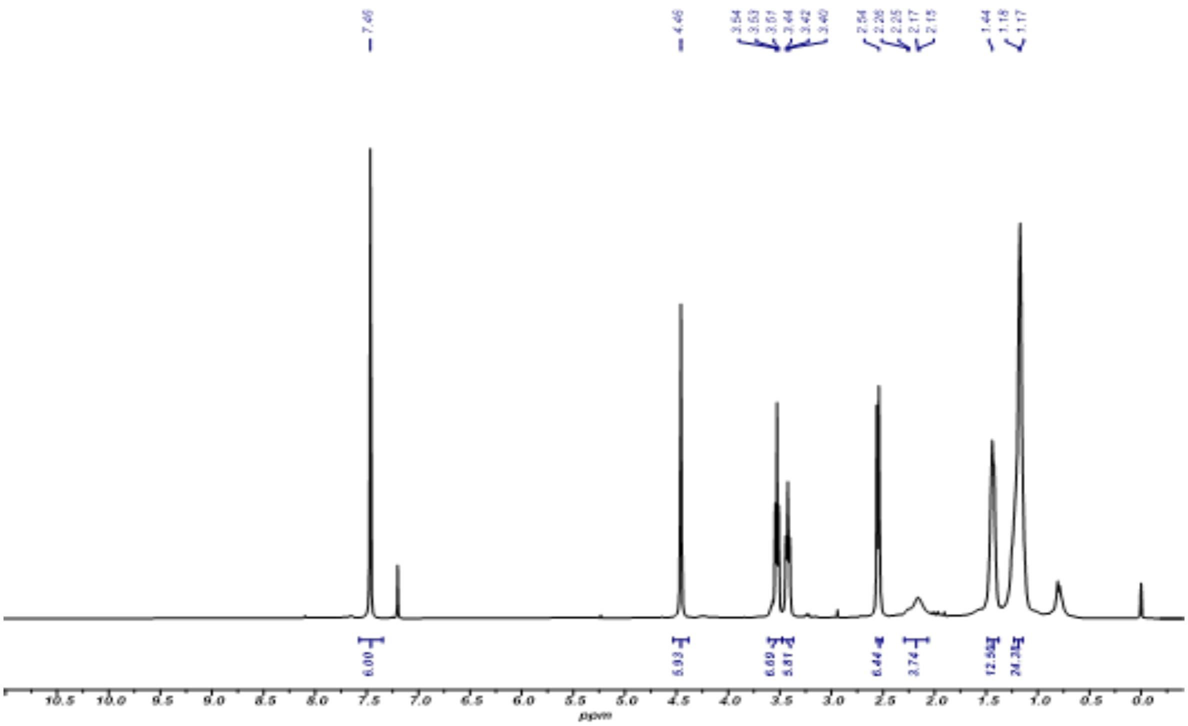

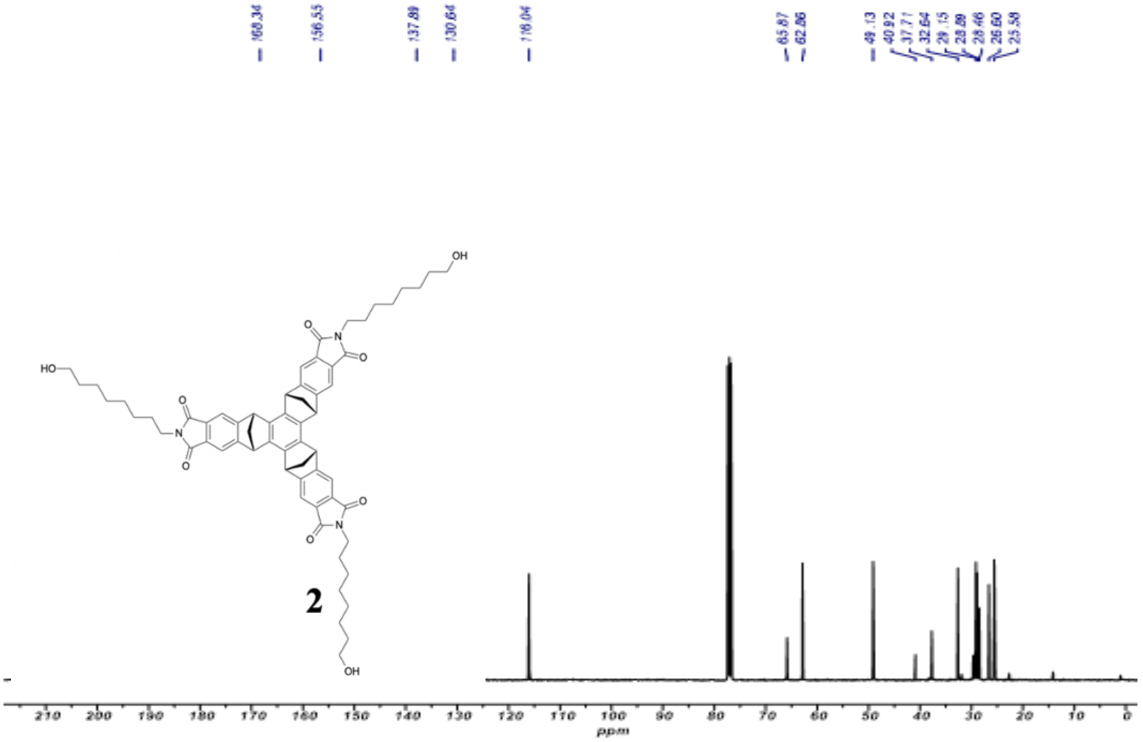

^{1}H NMR (top) and ^{13}C NMR (bottom) spectra of compound 2 (see page S2 for more info).

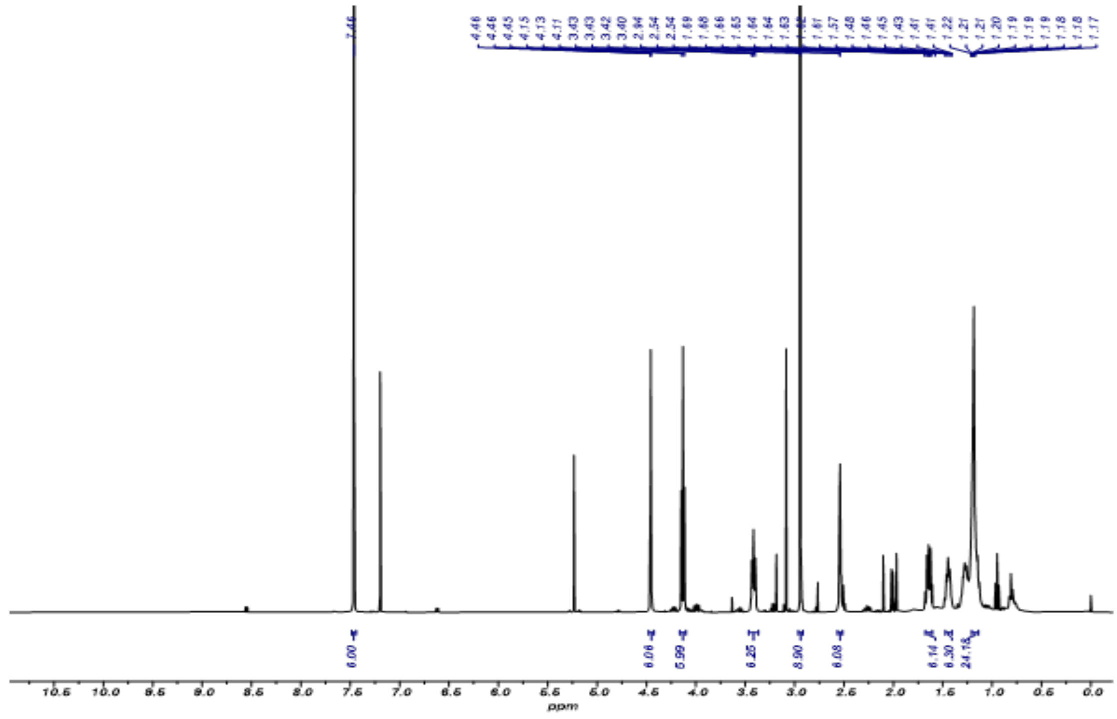

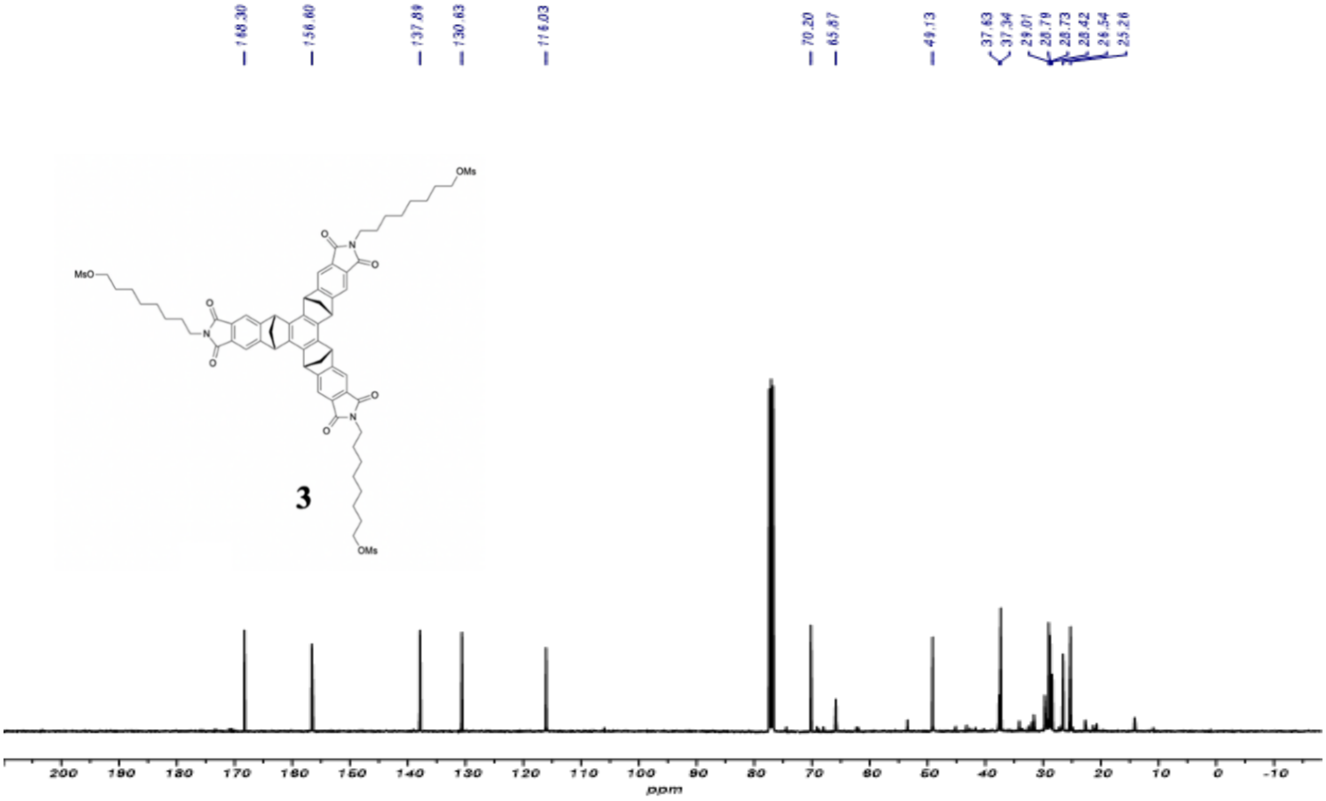

^{1}H NMR (top) and ^{13}C NMR (bottom) spectra of compound 3 (see page S3 for more info).

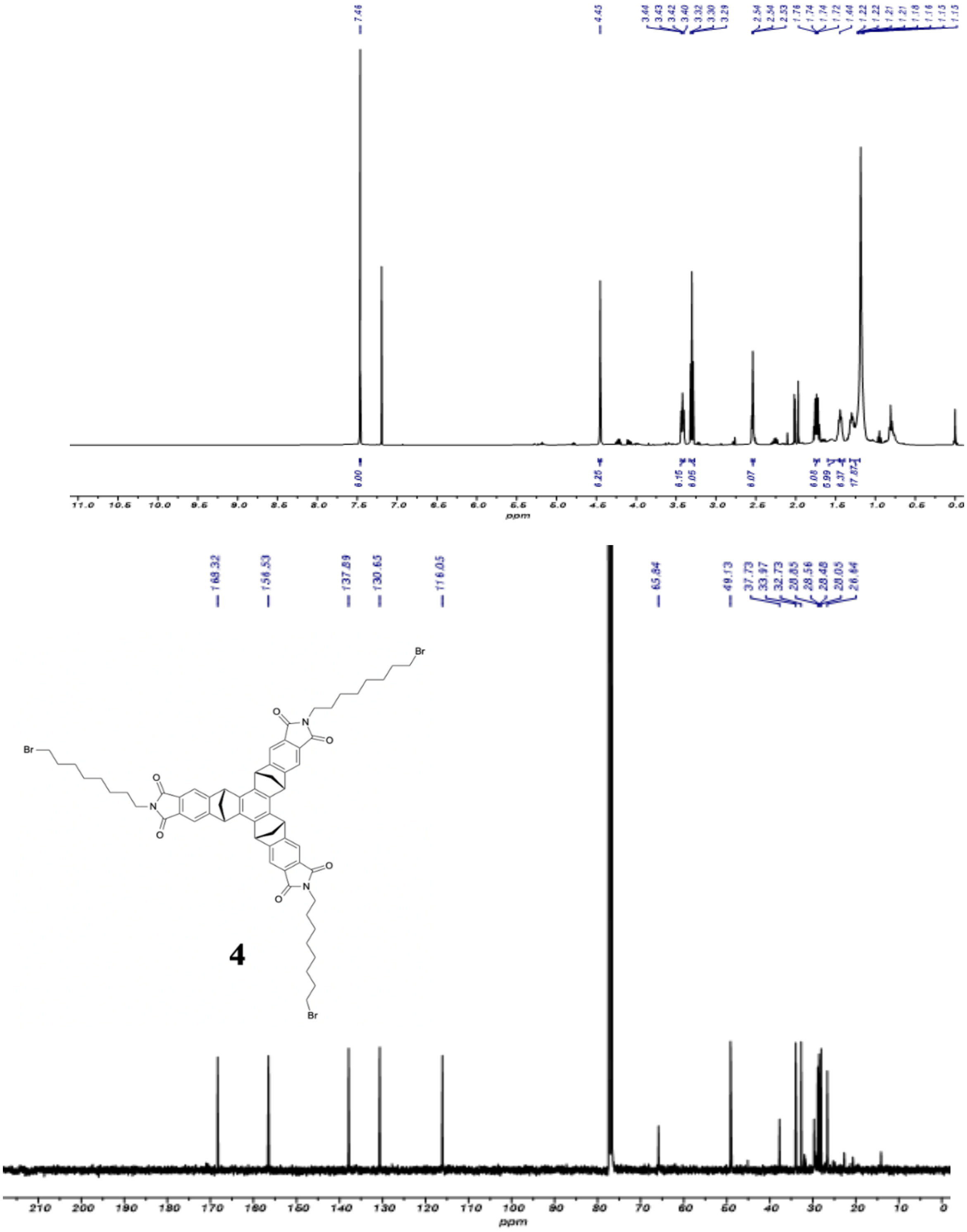

^{1}H NMR (top) and ^{13}C NMR (bottom) spectra of compound 4 (see page S3 for more info)

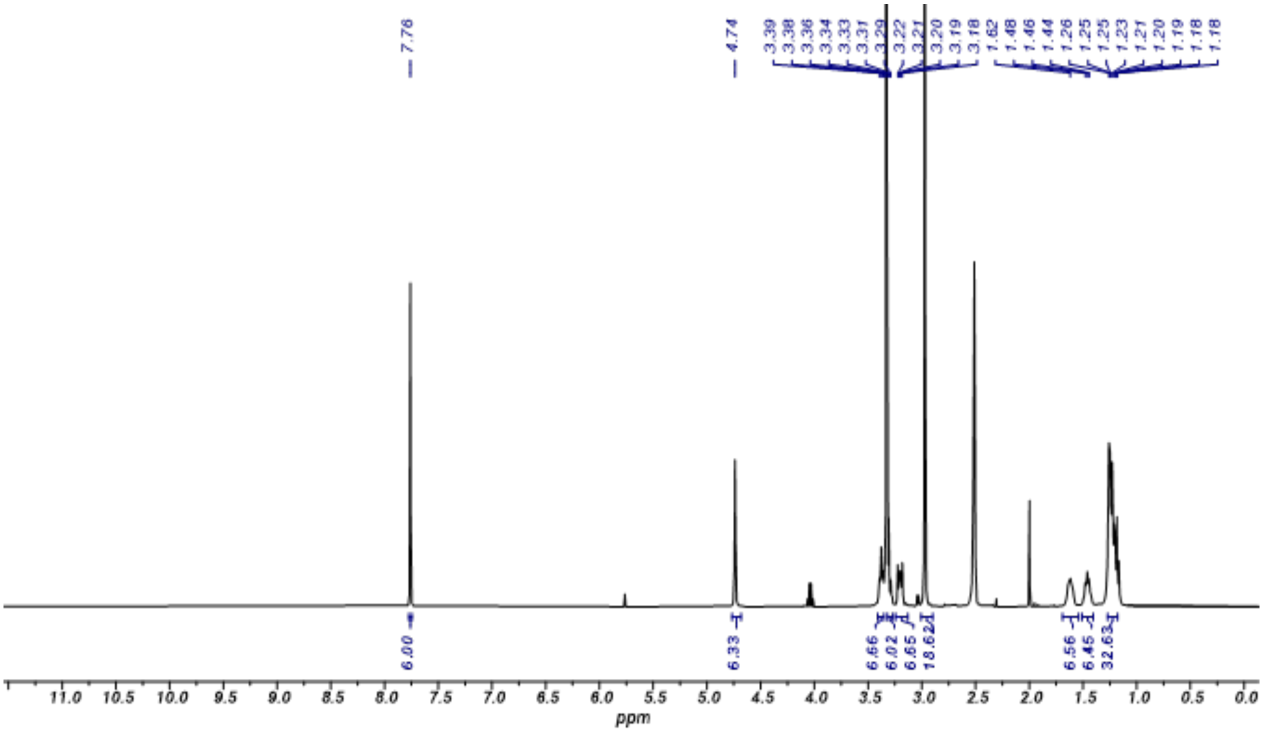

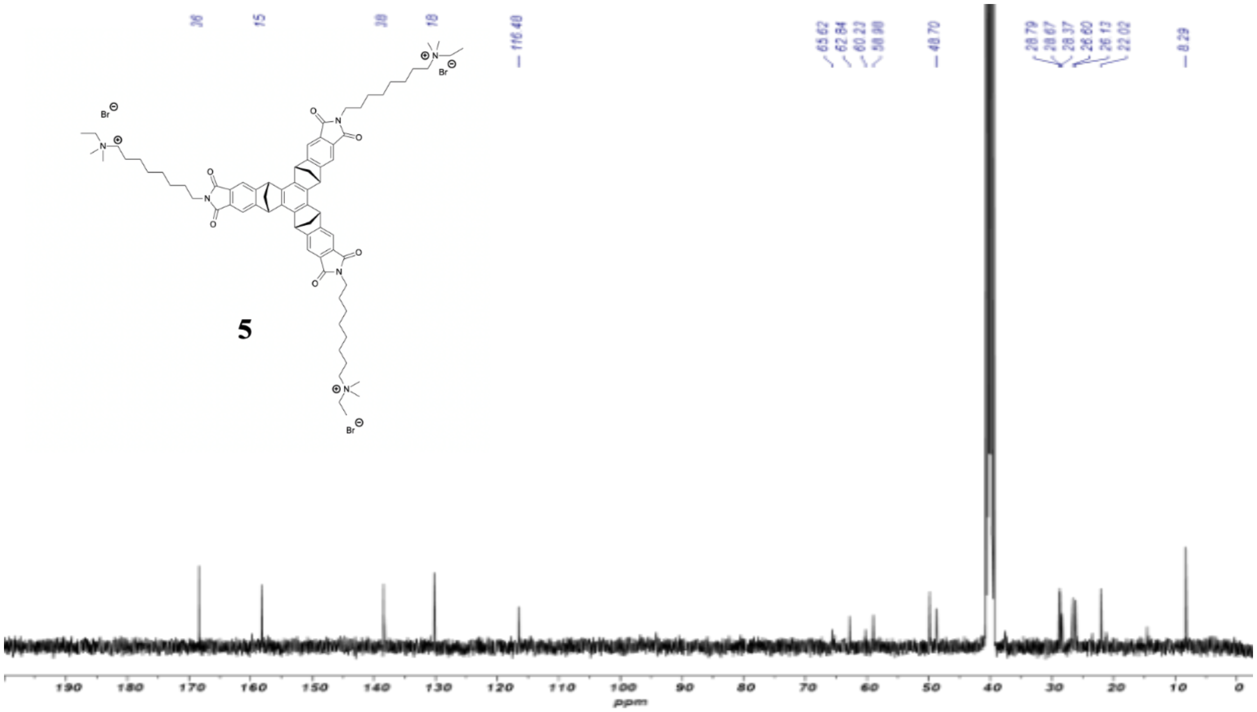

^{1}H NMR (top) and ^{13}C NMR (bottom) spectra of compound 5 (see page S3 for more info).

Amphiphilic Supramolecular Basket 12 (ASB-12)

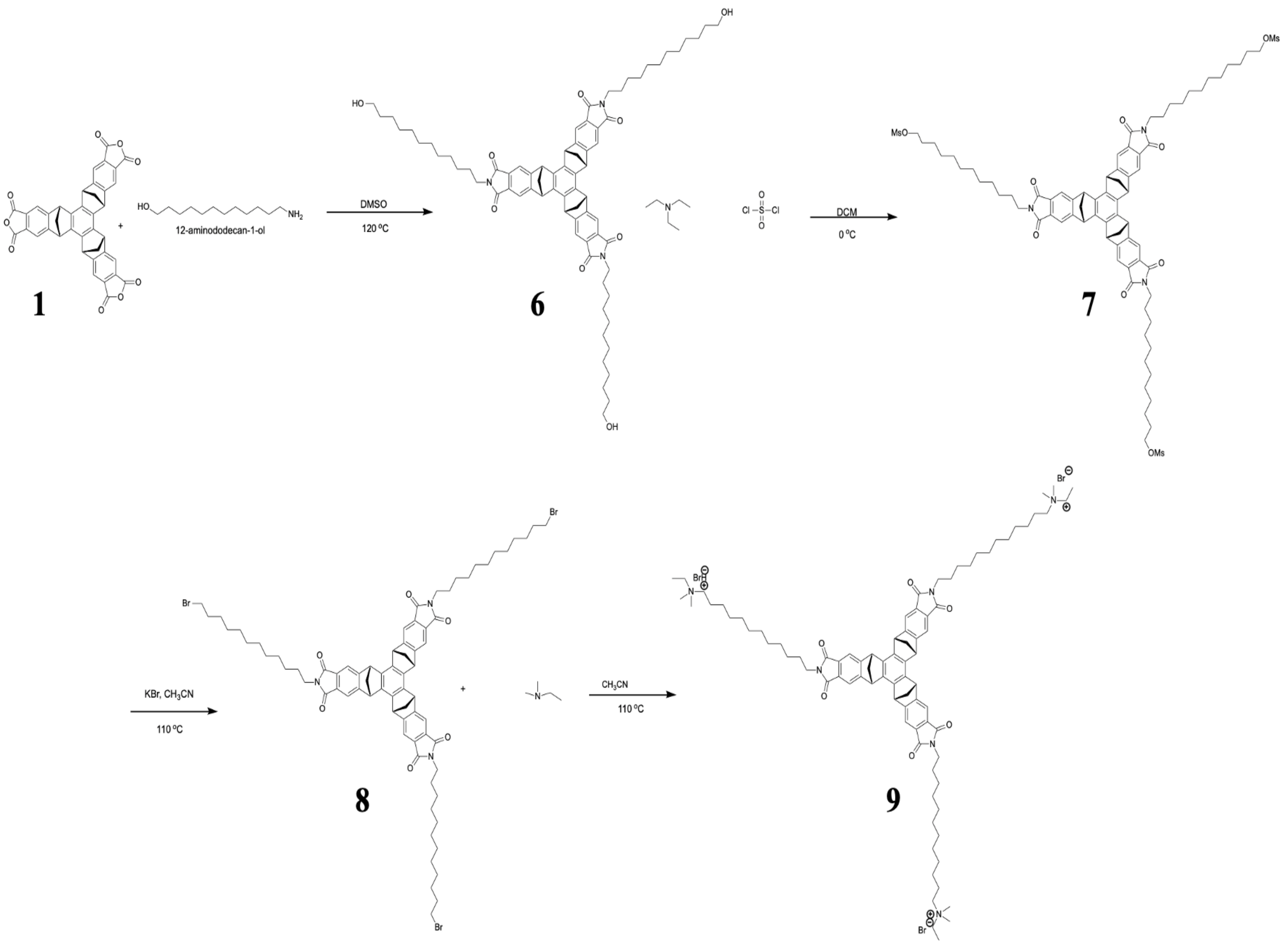

Scheme S2. Synthesis of ASB-12 (9). Supporting NMR data below.
For the preparation of 1, see Badjic et al. Molecules 2014, 19(9), 14292--14303.

Compound 6: To a suspension of tris-anhydride (1) (50 mg, 0.079 mmol) in DMSO (2 mL) was added 12-aminododecan-1-ol (47.93 mg, 0.23788 mmol). The mixture was heated at 120°C for 4 hours and then solvent was removed by using a strong stream of nitrogen gas. The solid residue was purified by column chromatography (SiO2, CH2Cl2:CH3OH=30:1) to yield SBTN-(CH2)12-OH (30 mg, 32%) 1H NMR (400 MHz, CDCl3) δ 7.54 (s, 6H), 4.53 (s, 6H), 3.64 (td, J = 6.6, 0.7 Hz, 6H), 3.49 (dd, J = 8.2, 6.4 Hz, 6H), 2.64 (s, 6H), 2.04 (s, 3H), 1.63 – 1.42 (m, 12H), 1.36 – 1.26 (m, 48H).13C NMR (101 MHz, CDCl3) δ 168.32, 156.52, 137.88, 130.65, 116.03, 65.83, 62.99, 49.12, 40.96, 37.80, 32.82, 29.53, 29.45, 29.41, 29.37, 29.05, 28.54, 26.77, 25.77. HRMS ESI: m/z calculated for C75H93N3O9:1179.64 [M+Na]+,found: 1202.68

Compound 7: To a solution of SBTN-(CH2)12-OH (30 mg, 0.049 mmol) in dry dichloromethane (5.0 mL) at 0°C was added triethylamine (60 mg, 0.593 mmol) and methanesulfonyl chloride (34 mg, 0.296 mmol). The mixture was stirred for 4 hours and water (10.0 mL) was added. After dilution with dichloromethane (30 mL), the organic phase was washed with water (20 mL) and brine (20 mL),and dried over sodium sulfate. Upon removal of the solvent, the solid residue was purified by column chromatography (SiO_2, CH_2Cl_2:CH_3OH = 100:1) to yield SBTN-(CH2)12-OMs (25 mg, 83%). 1H NMR (400 MHz, $CDCl_3$) δ 7.47 (s, 6H), 4.76 – 4.38 (m, 6H), 4.16 (t, J = 6.6 Hz, 6H), 3.62 – 3.23 (m, 6H), 2.95 (s, 9H), 2.54 (s, 6H), 1.67 (dt, J = 8.4, 6.6 Hz, 6H), 1.43 (t, J = 7.0 Hz, 6H), 1.23 – 1.15 (m, 48H). 13C NMR (101 MHz, $CDCl_3$) δ 168.30, 156.57, 137.89, 130.64, 116.03, 70.33, 65.86, 49.12, 45.98, 39.43, 37.77, 37.36, 29.43, 29.41, 29.37, 29.13, 29.04, 29.02, 28.53, 26.75, 25.43, 8.64. HRMS ESI: m/z calculated for $C_{78}H_{99}N_3O_{15}S_3$:1413.62 [M+H]+,found: 1414.63

Compound 8: To a solution of SBTN-(CH2)12-OMs (25 mg, 0.040 mmol) in dry acetonitrile (5.0 mL) was added KBr (477 mg, 4.0 mmol). The mixture was heated in a sealed tube at 110°C for 10 hours. Solvent was removed under reduced pressure and solid residue was dissolved in dichloromethane (20 mL) and water (20 mL). The organic layer was washed with brine (30 mL) and dried over sodium sulfate. Upon removal of the solvent, the solid residue was purified by column chromatography (SiO_2, CH_2Cl_2:CH_3OH=80:1) to yield SBTN-(CH2)12-Br (20 mg, 80%). 1H NMR (400 MHz, $CDCl_3$) δ 7.46 (s, 6H), 4.45 (s, 6H), 3.54 – 3.36 (m, 6H), 2.53 (t, J = 1.7 Hz, 6H), 1.85 – 1.72 (m, 6H), 1.43 (t, J = 7.1 Hz, 6H), 1.33 (dt, J = 8.8, 4.4 Hz, 6H), 1.27 – 1.15 (m, 48H). 13C NMR (101 MHz, $CDCl_3$) δ 168.26, 156.46, 137.88, 130.70, 116.01, 65.89, 49.14, 37.81, 34.10, 32.88, 29.09, 28.78, 28.57, 28.22, 26.80. HRMS ESI: m/z calculated for $C_{75}H_{90}Br_3N_3O_6$:1367.44 [M+H]+,found:1368.38

Compound 9: To a solution of SBTN-(CH2)12-Br (20.0 mg, 0.0333 mmol) in dry acetonitrile (10.0 mL) was added N,N-dimethylethylamine (243 mg, 3.33 mmol). The mixture was heated in a sealed tube at 110°C for 10 hours, and then concentrated under reduced pressure. The solid residue was recrystallized from CH_3OH:ethyl acetate=1:10 to yield SBTN-(CH2)12-NEtMe2Br (15 mg, 75%). 1H NMR (400 MHz, CD_3CN) δ 7.65 (s, 6H), 4.71 (s, 6H), 3.43 (t, J = 7.2 Hz, 6H), 3.31 (t, J = 7.3 Hz, 6H), 3.22 – 3.12 (m, 6H), 2.95 (d, J = 2.0 Hz, 18H), 1.69 (s, 6H), 1.50 (s, 6H), 1.38 – 1.27 (m, 63H). 13C NMR (101 MHz, CD_3CN) δ 168.80, 158.13, 139.01, 131.07, 117.89, 116.29, 65.93, 60.01, 50.48, 49.32, 37.98, 29.77, 29.70, 29.66, 29.58, 29.35, 29.29, 28.71, 27.00, 26.43, 22.62, 8.11, 1.52, 1.46, 1.31, 1.26, 1.11, 1.05, 0.90, 0.84, 0.69, 0.49, 0.28. HRMS ESI: m/z calculated for $C_{87}H_{122}N_3O_9$:448.98 [M-3Br]3+,found: 449.32.

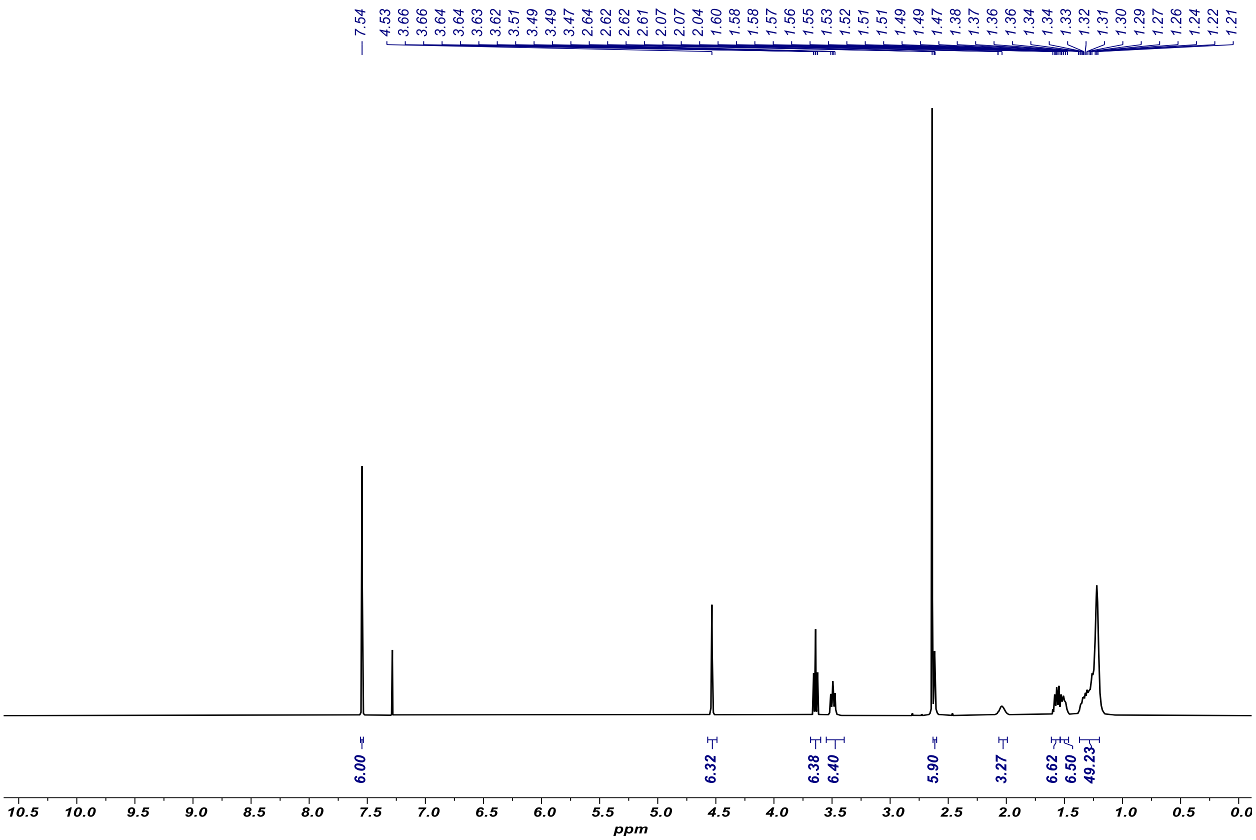

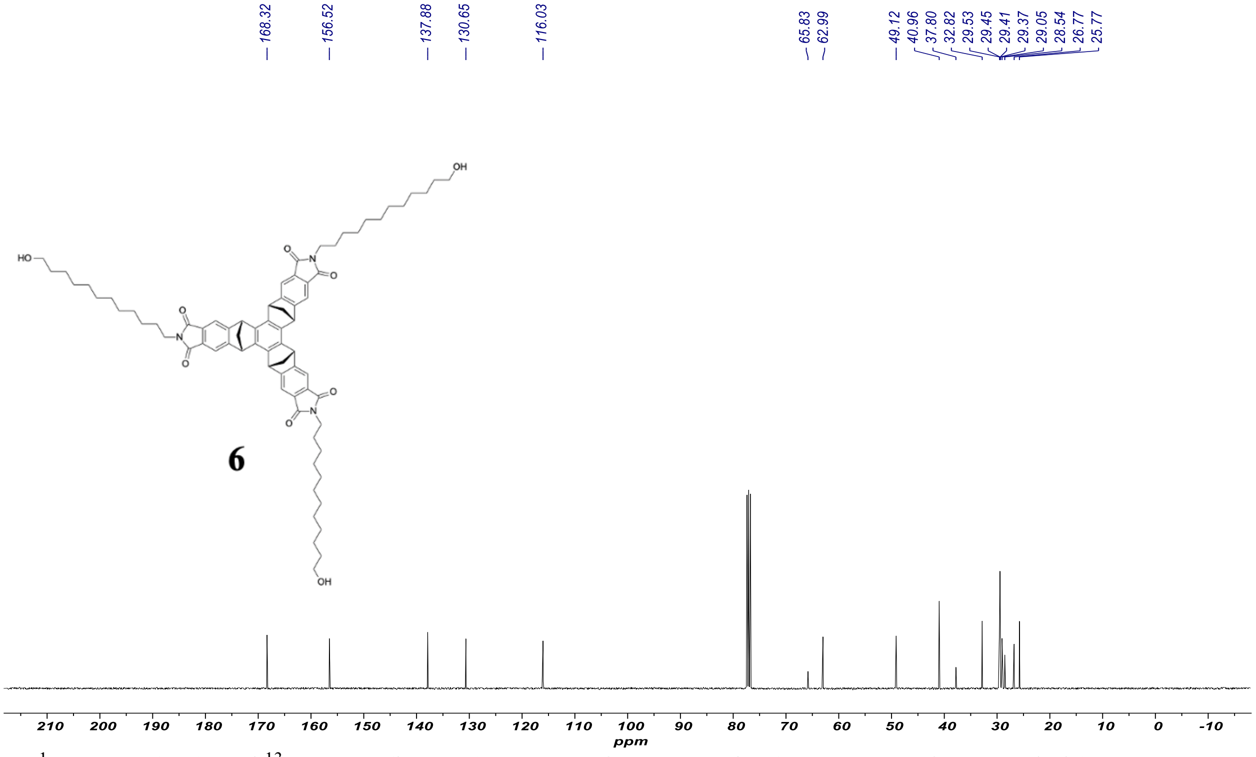

^{1}H NMR (top) and ^{13}C NMR (bottom) spectra of compound 6 (see page S9 for more info).

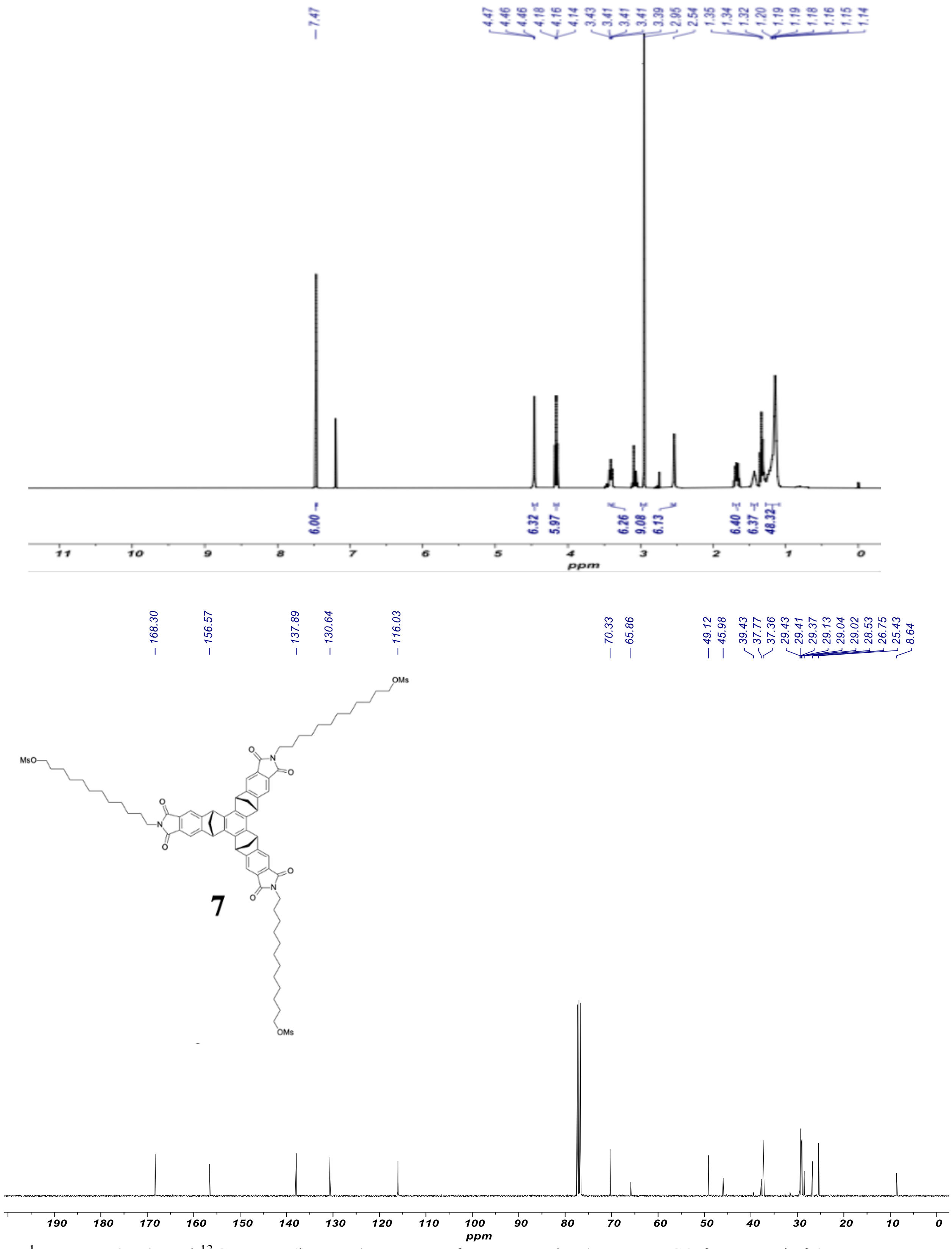

^{1}H NMR (top) and ^{13}C NMR (bottom) spectra of compound 7 (see page S9 for more info).

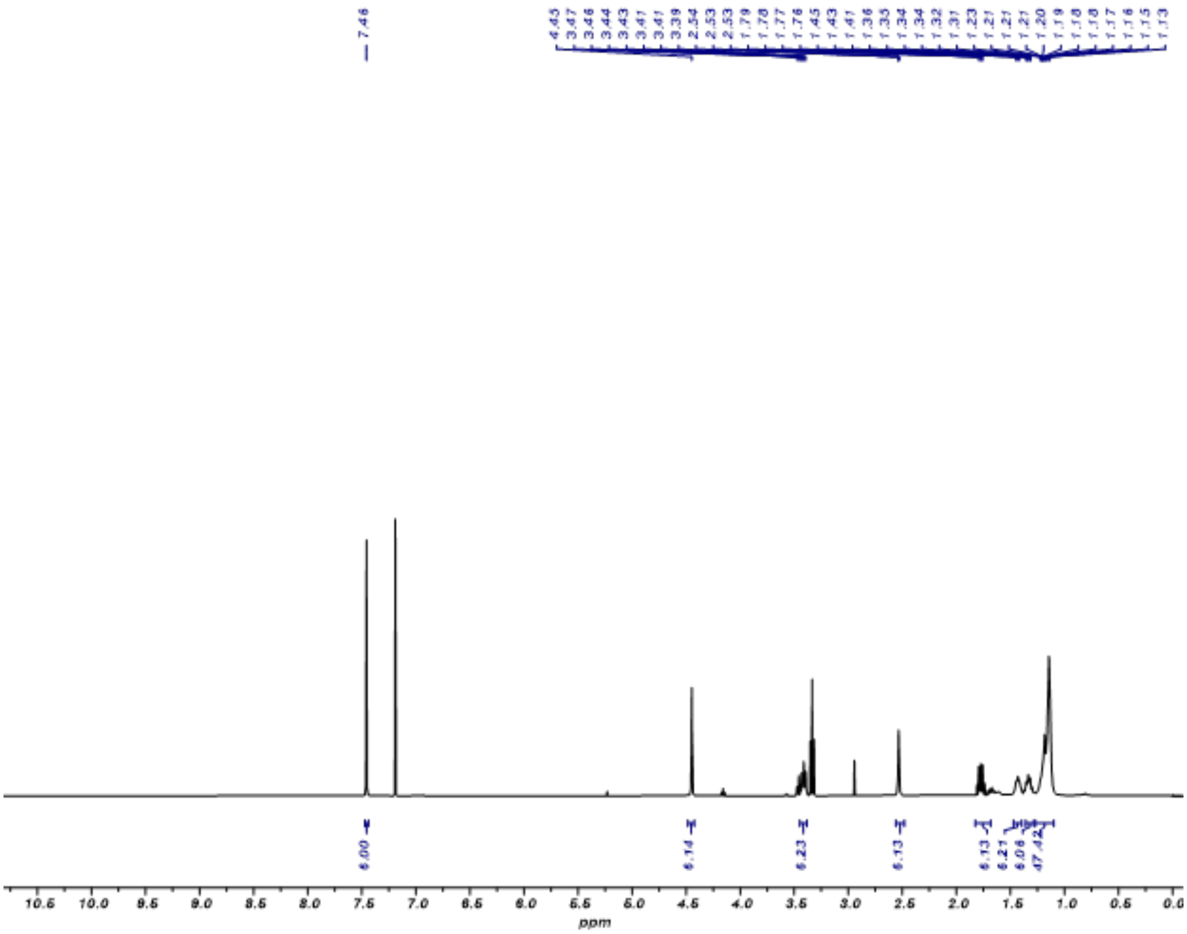

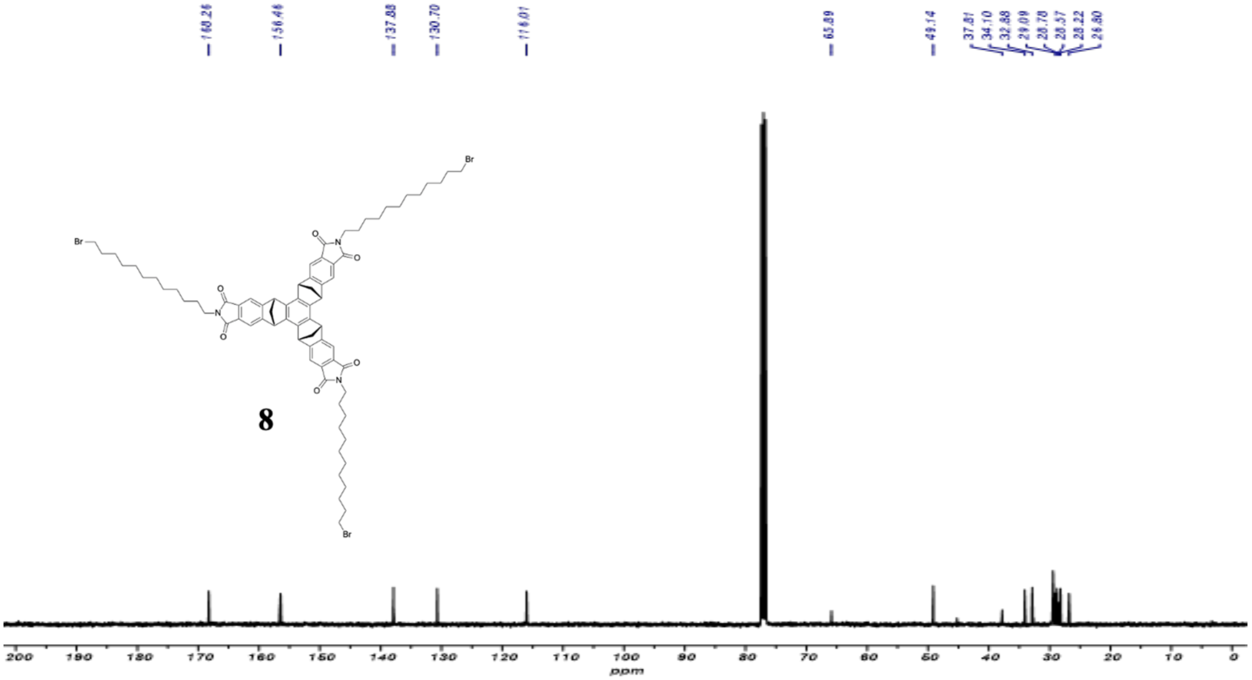

^{1}H NMR (top) and ^{13}C NMR (bottom) spectra of compound 8 (see page S9 for more info).

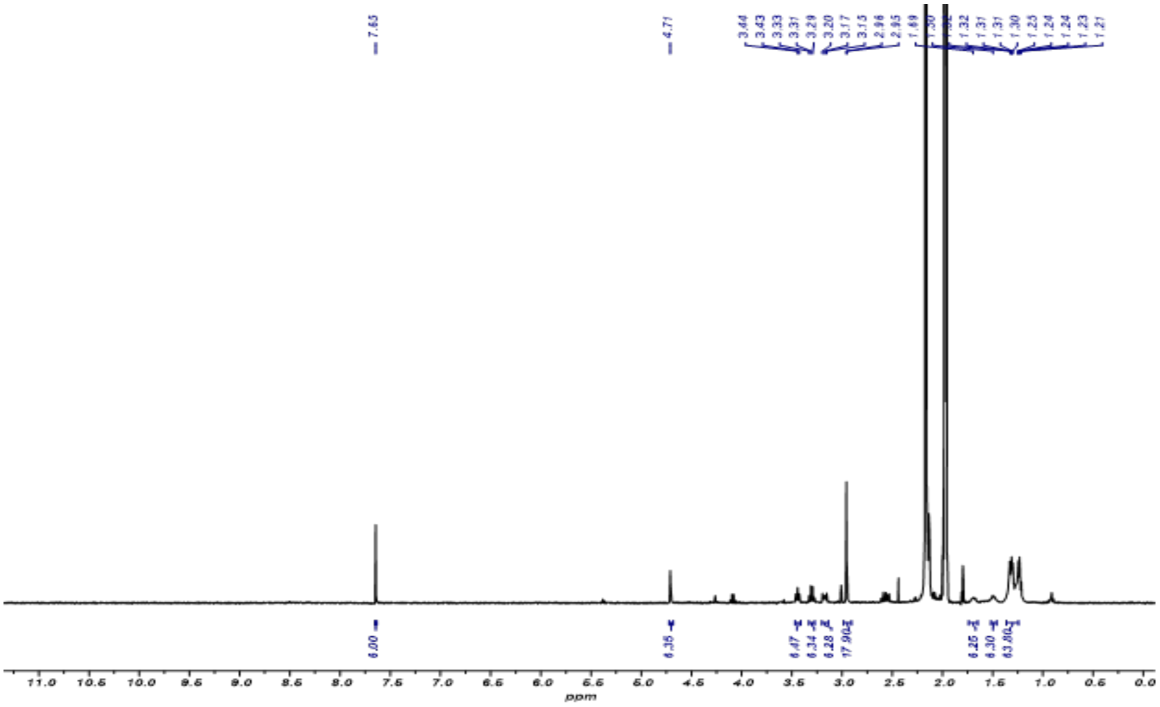

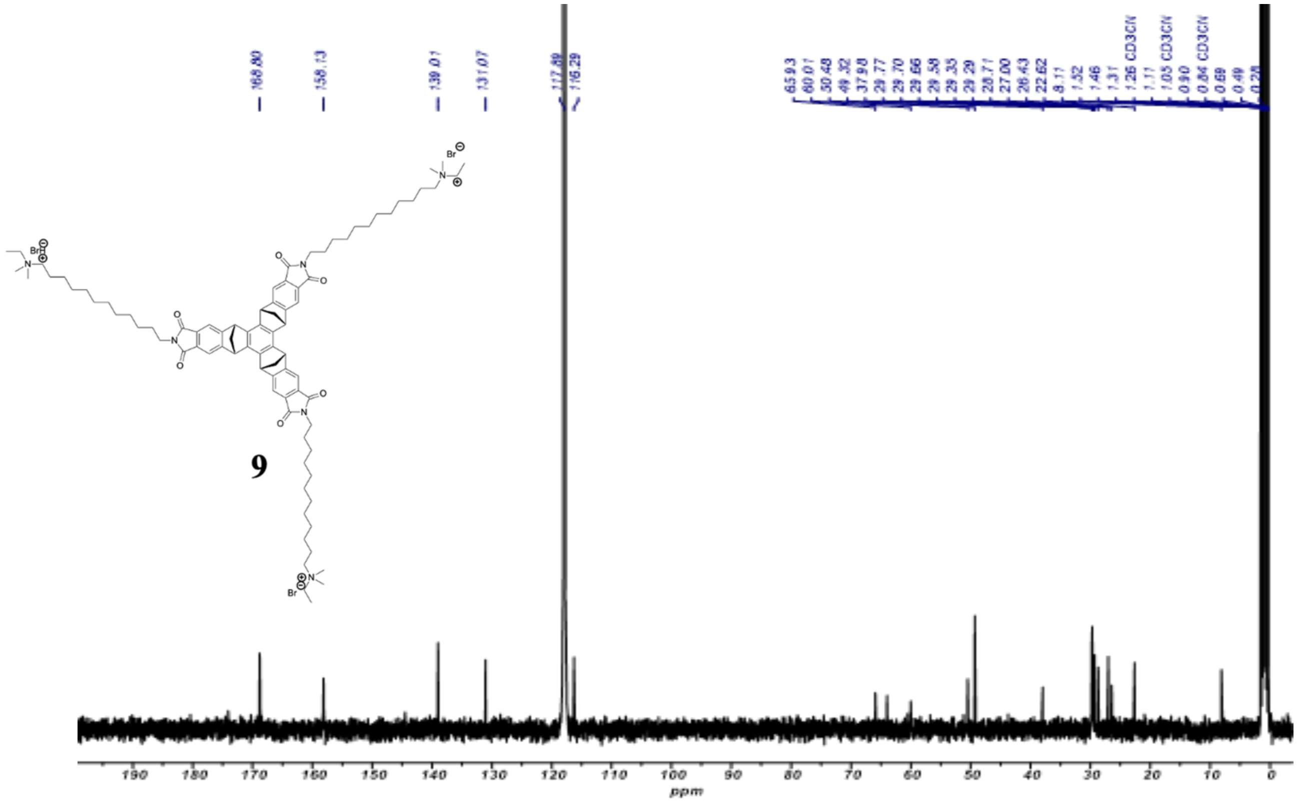

9

^{1}H NMR (top) and ^{13}C NMR (bottom) spectra of compound 9 (see page S9 for more info).

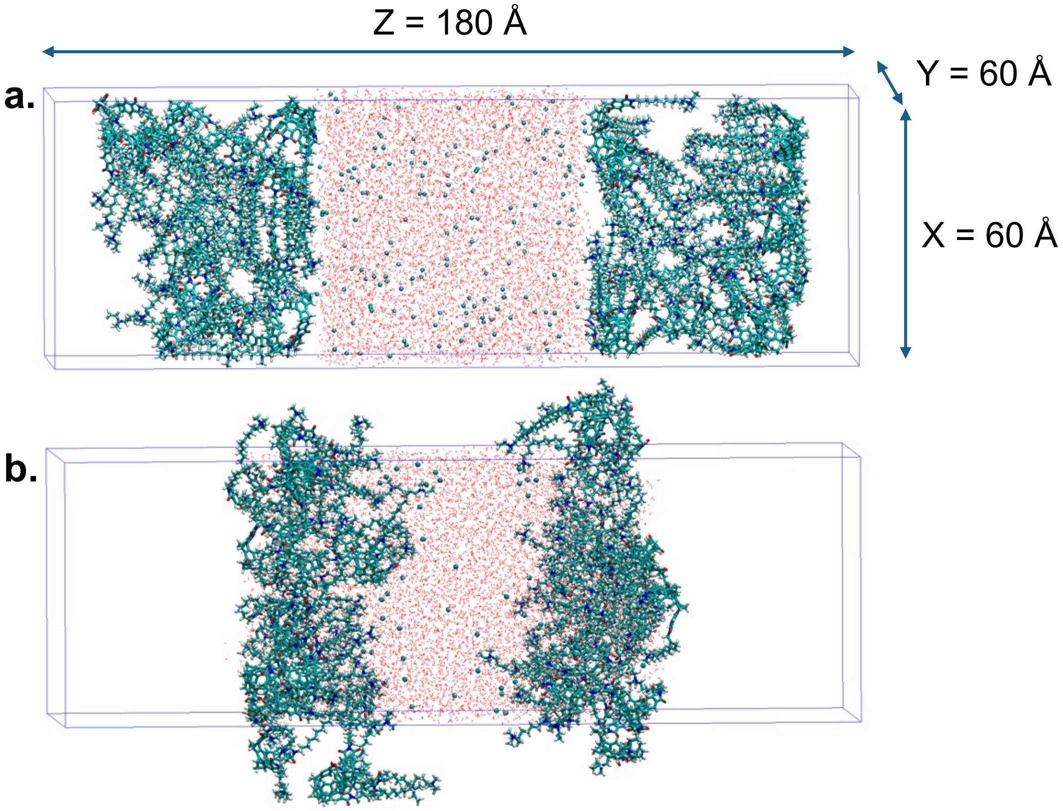

Figure S1. Representative snapshots of the molecular basket system (a) immediately after initialization and (b) 150 nanoseconds of simulation in the canonical ensemble. The panels illustrate the structural evolution from a dispersed configuration to an interfacial supramolecular layer formed by laterally associated baskets at the air water boundary.

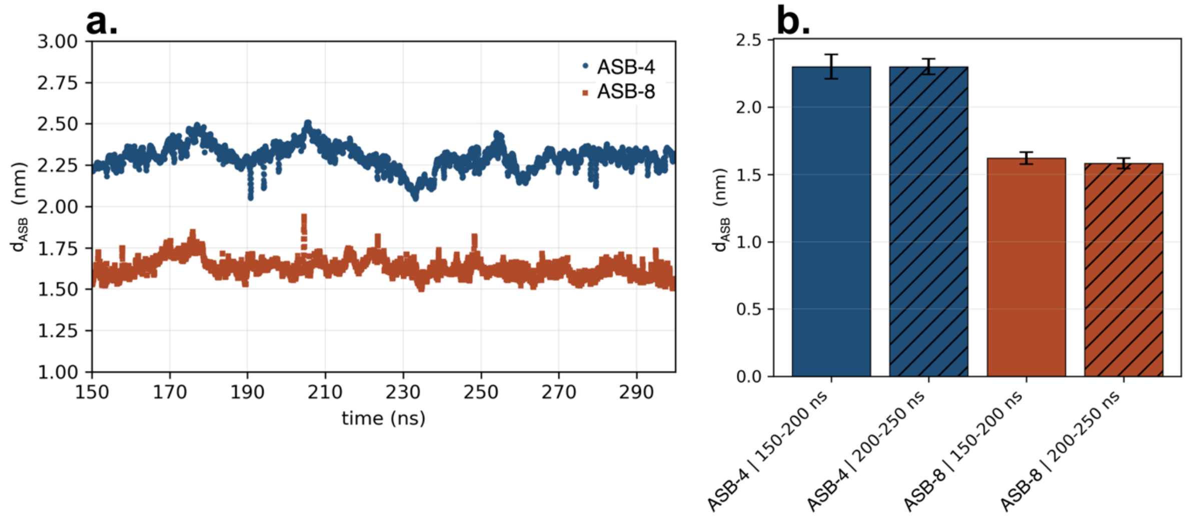

Figure S2. (a) Time evolution of the basket-to-nearest-Gibbs-dividing-surface distance, d_{ASB} for ASB-4 (blue) and ASB-8 (orange) systems. (b) Block-averaged d_{ASB} over consecutive 50 ns windows for the same two systems; error bars denote the within-block standard deviation.

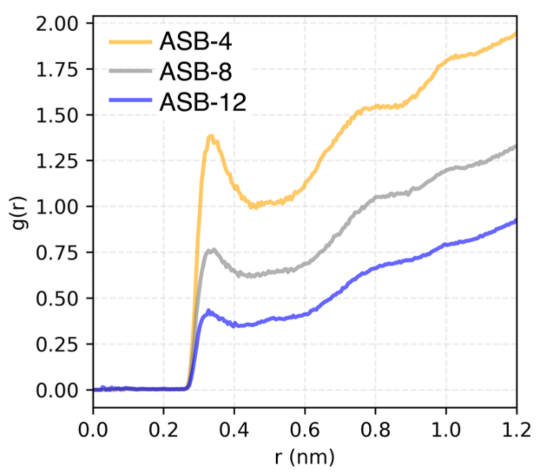

Figure S3. Pair correlation functions between water oxygen atoms (O_W) and basket oxygen atoms (O_{ASB}) for the ASB-4, ASB-8, and ASB-12 systems.

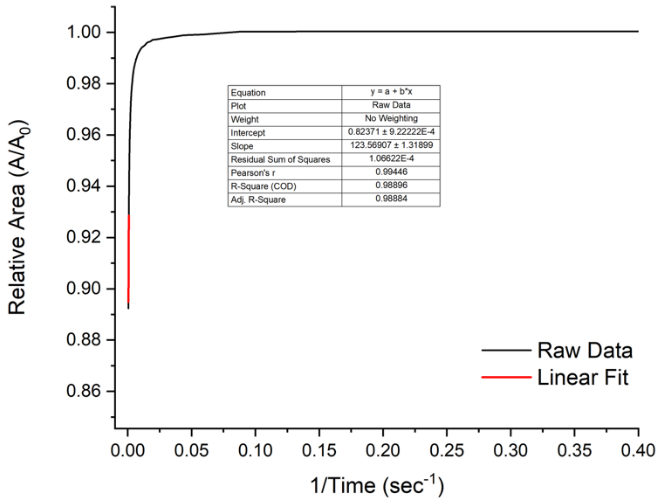

Figure S4. Relative area plotted against the reciprocal of time (1/Time) to determine A_∞. The y-intercept of the liner fit represents A_∞/A_0. Because A_0 is known, A_∞ can be calculated from the y-intercept.

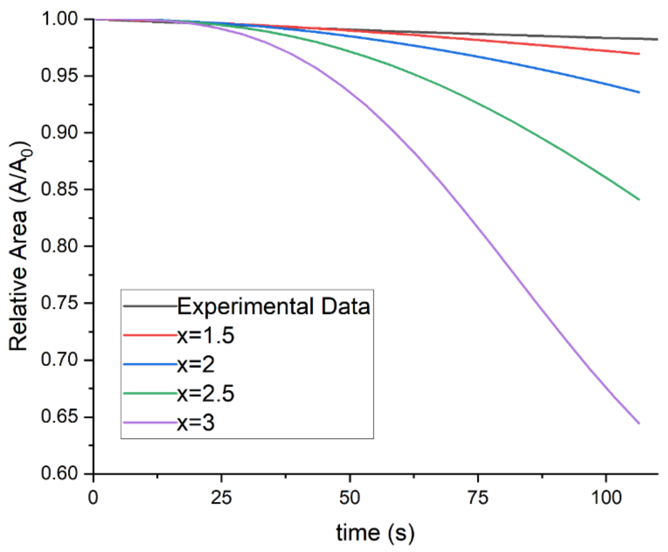

Figure S5. Variability in constant pressure relation of ASB-12 on water at 4.8mN/m. The ability of the system to maintain a 4.8mN/m surface pressure affects the relative area vs. time curve, and thus the characteristic parameter, x.

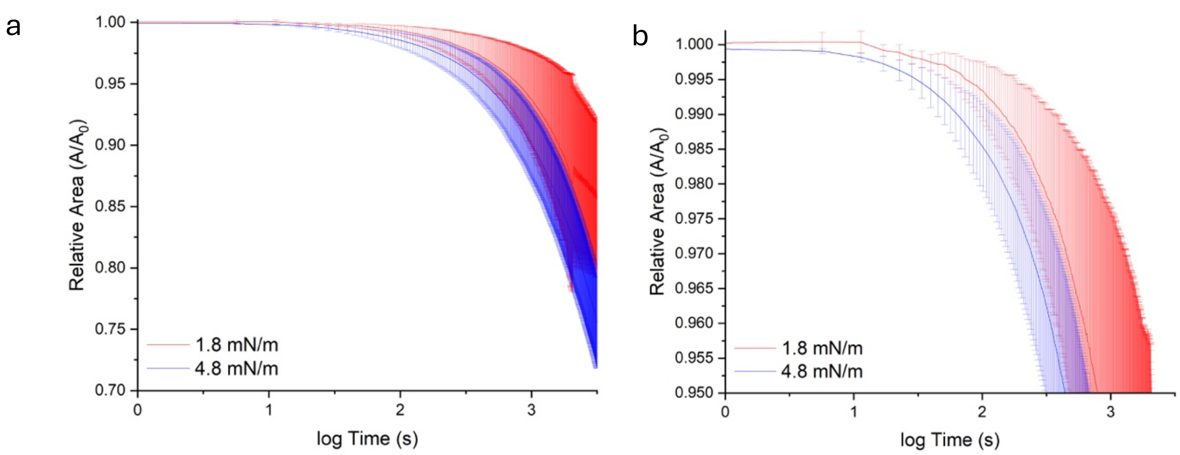

Figure S6. Semi-logarithmic representation of relative area relaxation for ASB-12 monolayers at the air–water interface. Relative area, A/A_0, is plotted as a function of time using a semi-logarithmic x-axis to better visualize the exponential decay behavior of the monolayer relaxation process. Data corresponds to the relaxation measurements shown in Figure 2b. The right figure (b) is an expanded view of the y-axis to better display the separation between the data sets.

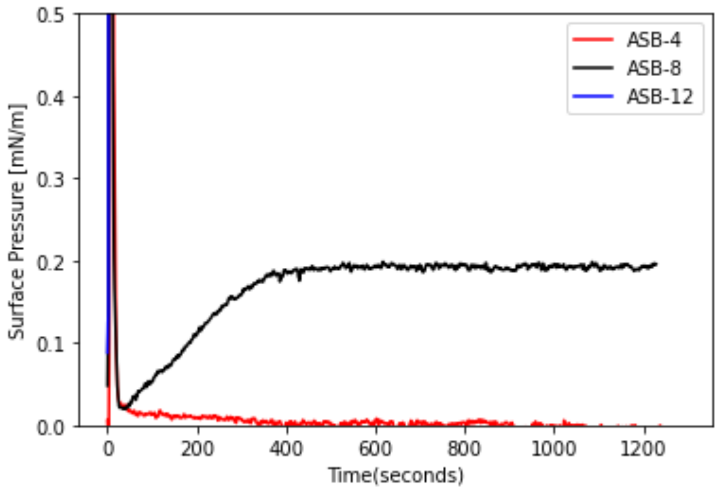

Figure S7. Time evolution of surface pressure curves of ASB-4 (red) and ASB-8 (black) on water spread. This experiment was conducted to ensure the stability of the film during IRRAS acquisition time. These systems did not show significant increased surface pressures. Each IRRAS spectra are taken over an interval of 7 minutes**.**

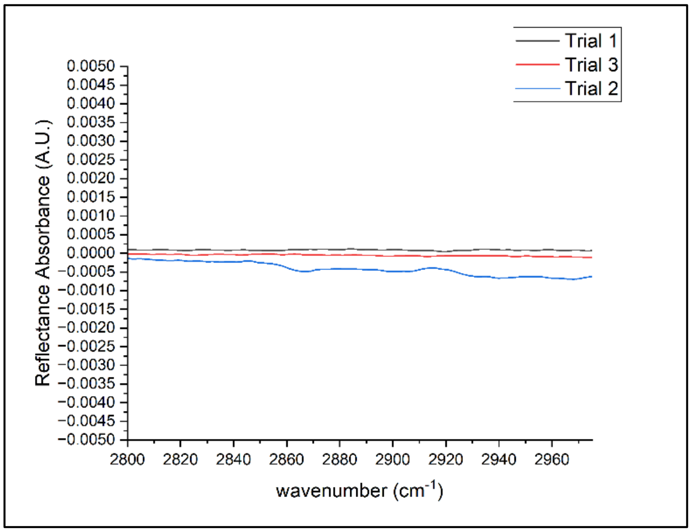

Figure S8. IRRAS spectra of alkyl region of ASB-4 (~2.13x 10^{13} molecules/cm^2) monolayers on water. Plotted here are the triplicate data to ensure reproducibility.

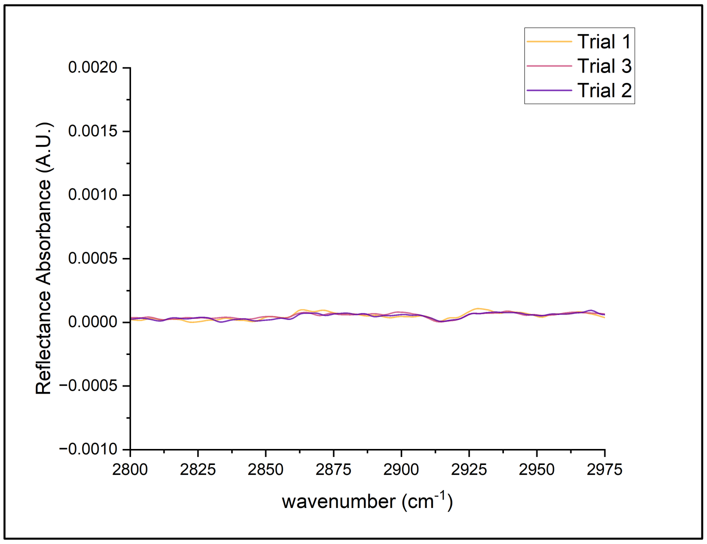

Figure S9. IRRAS spectra of alkyl region of ASB-8 (~2.13x 10^{13} molecules/cm^2) monolayers on water. Plotted here are the triplicate data to ensure reproducibility.

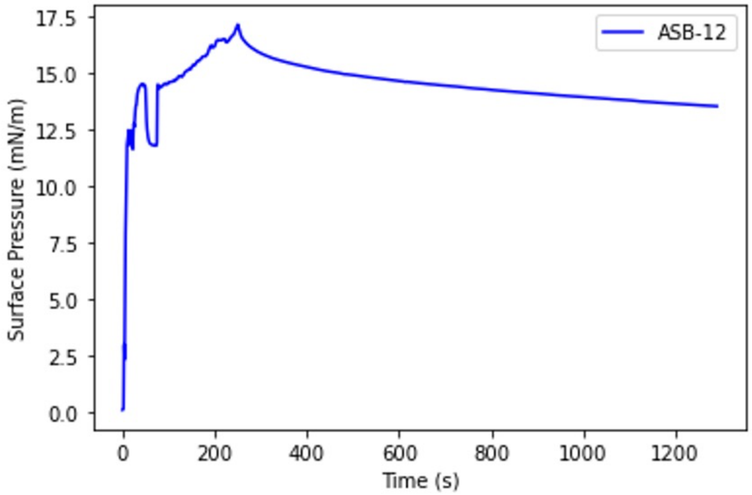

Figure S10. Time evolution of surface pressure curve of ASB-12. This experiment was conducted to ensure the stability of the film during IRRAS acquisition time. Each IRRAS spectra are taken over an interval of 7 minutes.

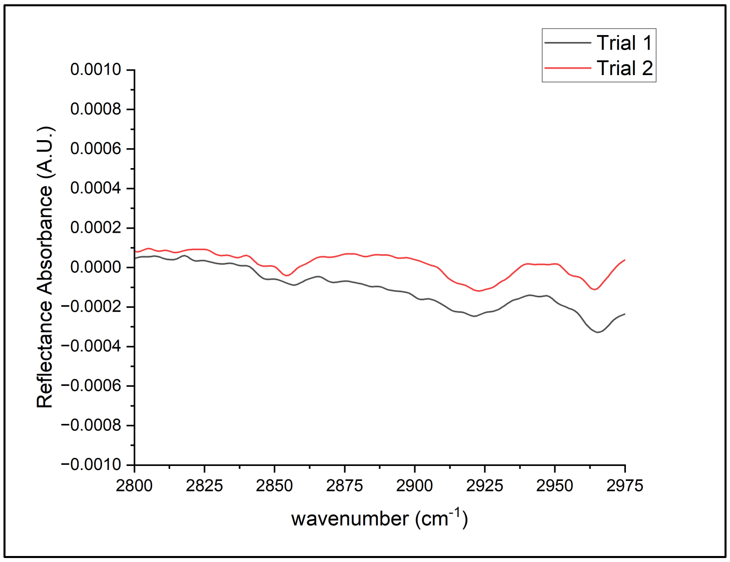

Figure S11. IRRAS spectra of ASB-12 (~2.13x 10^{13} molecules/cm^2) on pure water. Plotted here are the duplicate data to ensure reproducibility.

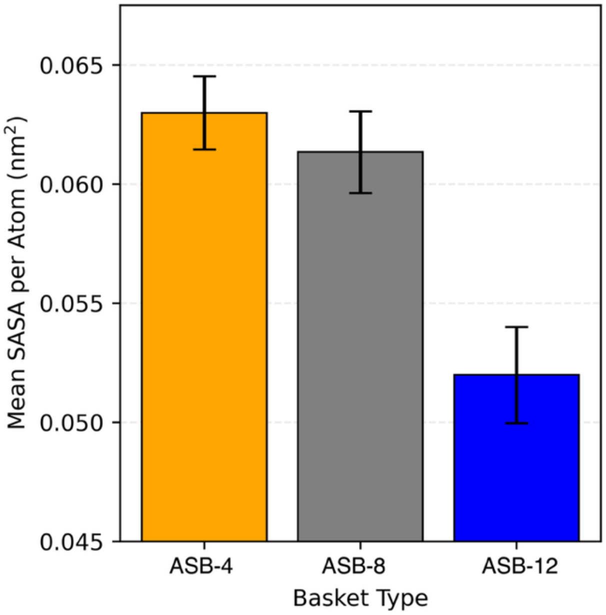

Figure S12. Mean solvent-accessible surface area (SASA) per atom of the molecular baskets, normalized by the number of atoms per basket for ASB-4 (orange), ASB-8 (grey), and ASB-12 (blue) systems. Error bars denote the trajectory-frame standard deviation.

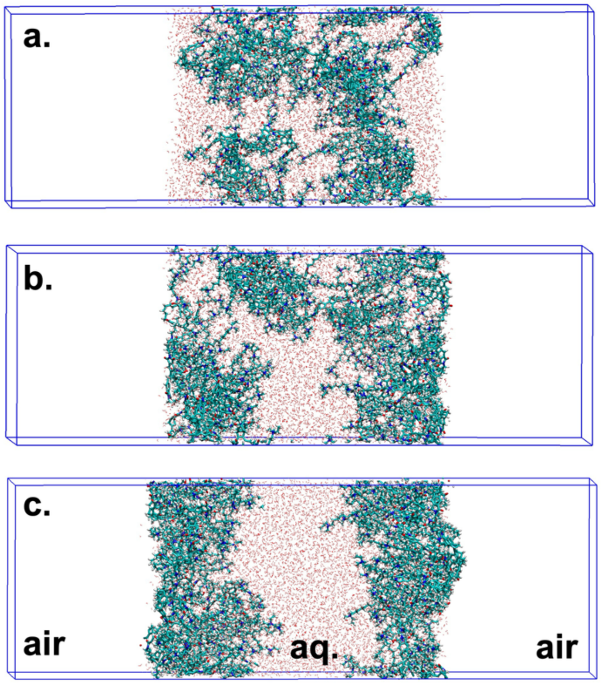

Figure S13. Molecular dynamics snapshots wrapped across periodic boundaries, showing the equilibrium distribution of amphiphilic molecular baskets with hydrophobic arms of length (**a**) four, (**b**) eight, and (**c**) twelve methylene units at the air-water interface.

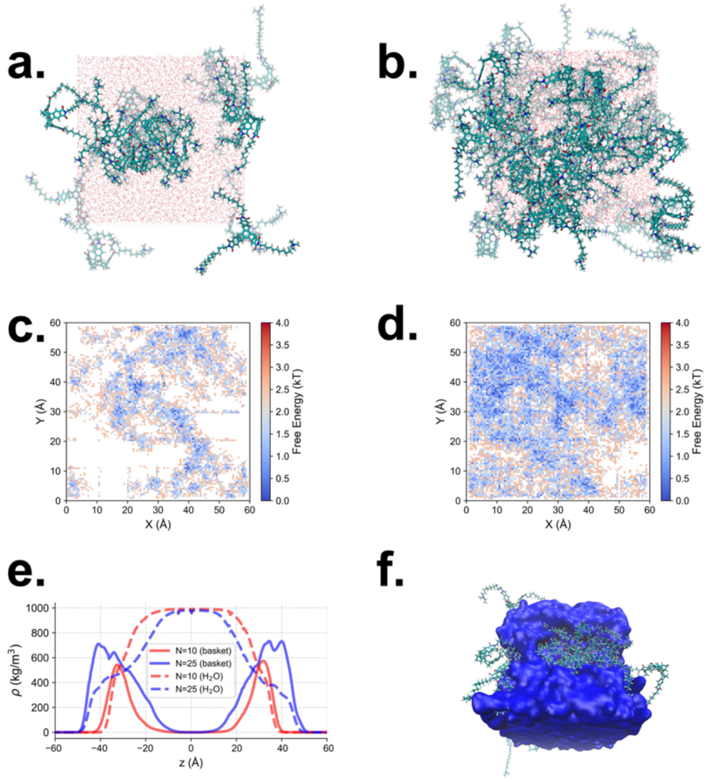

Figure S14. Structural and thermodynamic signatures of concentration dependent self-assembly and water displacement for the longest tail ASB-12 baskets. Panels (a) and (b) show top views of the simulation cell after equilibration for systems containing 10 and 25 baskets per interface, respectively, highlighting the lateral clustering behavior within the interfacial plane. Panels (c) and (d) present two-dimensional free energy landscapes for basket adsorption at the air water interface for systems with 10 and 25 baskets per interface, respectively. Panel (e) compares one-dimensional density profiles of baskets and water along the direction normal to the interface for the two concentrations, revealing an inverse relation between basket adsorption and interfacial water density. Panel (f) shows a top view of the simulation cell with water rendered as an iso-surface to visualize the formation of nanoscopic cavities within the interfacial region that arise from displacement of water from the interface into the bulk aqueous phase upon basket adsorption.

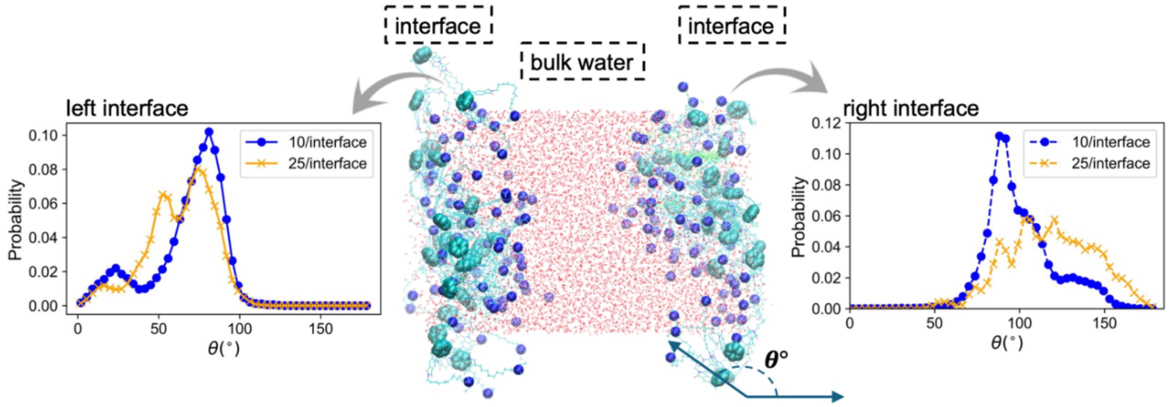

Figure S15. Probability distributions of basket orientation at the two air-water interfaces. Orientation is quantified by the angle between the interface normal and a vector connecting the center of mass of the central aromatic ring to the terminal tail nitrogen. Results are shown for ASB-12 systems with ten and twenty-five baskets per interface, highlighting the concentration dependent transition from a relatively narrow orientational ensemble that points toward the aqueous phase to a broader and more heterogeneous distribution at higher basket loading.

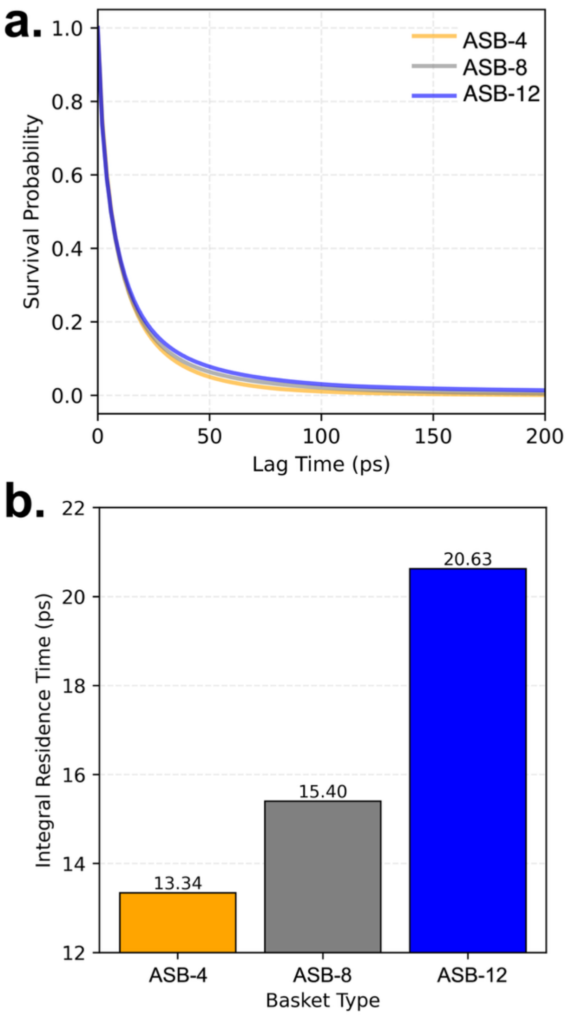

Figure S16. Translational dynamics of water within the first solvation shell of oxygen atoms (O_{ASB}) of ASB-4 (orange), ASB-8 (gray), and ASB-12 (blue). The solvation shell is defined by a 0.45 nm cutoff, corresponding to the first minimum of the O_{ASB} –O_W pair correlation function. (a) Continuous shell survival probability. (b) Corresponding integral residence times.

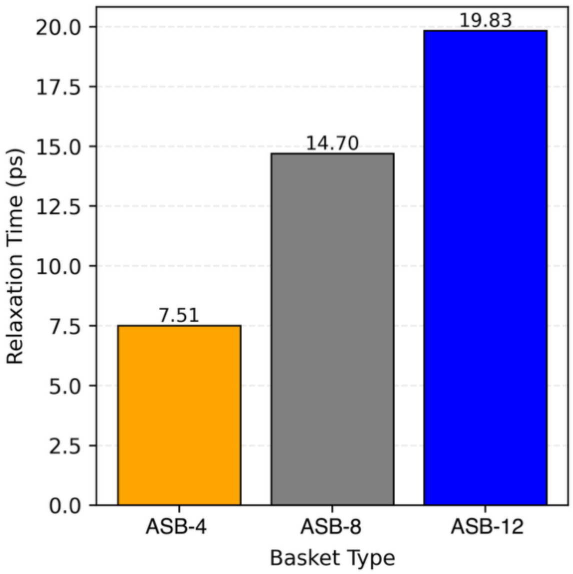

Figure S17. Orientational relaxation time of water within the first solvation shell of basket oxygen atoms O_{ASB} of ASB-4 (orange), ASB-8 (grey), and ASB-12 (blue).

Technique of Surface-Pressure Area Isotherm

Organic films that are one molecule thick can have structure that can be represented in various phases—gas, liquid, and condensed.[1,2] Within the two-dimensional (2D) gaseous phase, there is minimal organization between the molecules at the surface. Once the system is compressed, a transition from gaseous to liquid condensed phase occurs. The liquid condensed phase is usually dictated by a "lift-off point", which is a point where the surface pressure that is above a few tenths of a mN/m.[4,5] This phenomenon is a result of the organic molecules occupying less area and having greater interactions with neighboring molecules. As a result the molecules' mean molecular area (MMA) decreases leading to more intermolecular interaction, between these interfacial compounds.[1,4] The liquid phase can have two distinct states, the liquid-expanded (LE) and the liquid-condensed (LC).[3] With continued compression, the molecules begin to enter the collapsed (C) phase, which is a destabilization of the two-dimensional films. As a result, three-dimensional structures can form, such as aggregates. [1,4] The strength of intermolecular interactions dictates the types of phases.[4] Changes in temperature and surface pressure can affect the molecules packing and stability in the phases.[1,2] The number of distinct phases depend on the type of surfactant.

References

1. Butt, H.; Graf, K.; Kappl, M. Physics and Chemistry of Interfaces, 3rd ed.; WILEY-VCH Verlag GmbH & Co. KGaA: Weinheim, Germany, 2013.
2. Mendelsohn, R. Infrared Reflection-Absorption Spectroscopy of Monomolecular Films in Situ at the Air/Water Interface. Spectroscopy of Biological Molecules: Modern Trends 1997, 597–598.
3. Mendelsohn, Richard, et al. "External Infrared Reflection Absorption Spectrometry of Monolayer Films at the Air-Water Interface." Annual Reviews, vol. 46, no. 1, 1 Oct. 1995, pp. 305–334.
4. Gaines, G. L. Insoluble Monolayers at Liquid-Gas Interface; Interscience Publishers, 1966.
5. Knobler, Charles, and Rashmi Desai. "Phase Transitions in Monolayers." Annual Review Physical Chemistry, vol. 43, no. 1, Aug. 1992, pp. 207–236.
